# Development of a multi-targeted metabolomics platform for semi-quantification of faecal metabolites: a proof-of-concept analysis in human faeces

**DOI:** 10.20517/mrr.2025.85

**Published:** 2026-03-26

**Authors:** Kayo Ikuta, Akihiro Kunisawa, Itaru Dekio, Arisa Ito, Qiuyi Wang, Kazuhiro Kawamura, Masaki Yamada, Sumi Nakamura, Yoshihiro Hayakawa, Takuma Higurashi, Junko Iida, Eiichiro Fukusaki, Toru Suzuki, Mitsuharu Matsumoto

**Affiliations:** ^1^Dairy Science and Technology Institute, Kyodo Milk Industry Co. Ltd, Tokyo 190-0182, Japan.; ^2^Shimadzu Corporation, Kyoto 604-8511, Japan.; ^3^The University of Osaka and Shimadzu Analytical Innovation Research Laboratories, The University of Osaka, Osaka 565-0871, Japan.; ^4^Department of Dermatology, The Jikei University School of Medicine, Minato-ku, Tokyo 105-8461, Japan.; ^5^Department of Gastroenterology and Hepatology, Yokohama City University School of Medicine, Yokohama 236-0004, Japan.; ^6^Department of Biotechnology, Graduate School of Engineering, The University of Osaka, Osaka 565-0871, Japan.; ^7^BC LAB, Osaka 541-0047, Japan.; ^8^Leicester NIHR Biomedical Research Centre and BHF Centre of Research Excellence, University of Leicester, Leicester LE1 7RH, UK.; ^9^The Institute of Medical Science, The University of Tokyo, Tokyo 108-0071, Japan.; ^#^These authors contributed equally to this work.

**Keywords:** Intestinal microbiome, faeces, metabolome, triple quadrupole mass spectrometry, germ-free mice

## Abstract

**Objectives:** Targeted metabolomic analysis of faecal samples has been limited by narrow chemical coverage. Here, we established a multiplexed, triple quadrupole mass spectrometry (TQMS)-based targeted metabolomics workflow. This workflow allows accurate detection and semi-quantification of diverse faecal metabolites and provides a methodological platform for studying host-microbiome metabolic interactions.

**Methods:** Faecal metabolomes from germ-free (GF) mice, ex-germ-free (Ex-GF) mice, and human participants were analysed using TQMS-based targeted metabolomics. The analysis comprised multiple methods targeting amino acids and their derivatives, carbohydrates, short-chain fatty acids, bile acids, lipid mediators, and phospholipids.

**Results:** In total, 607 low-molecular-weight metabolites in 44 chemical categories were detected and semi-quantified. Faecal metabolomes of GF and Ex-GF mice were analysed, uncovering 341 intestinal microbiome-dependent metabolites. A proof-of-concept analysis using faecal samples from five patients with colorectal cancer demonstrated the successful application of this platform to human clinical material, highlighting its strong potential for future disease-oriented metabolomic investigations.

**Conclusion:** We developed a multi-targeted faecal metabolomics platform that substantially expands the chemical space accessible to targeted analysis. This workflow provides a methodological foundation for future large-scale and translational studies.

## INTRODUCTION

To optimise therapeutic strategies aimed at manipulating the intestinal microbiome to combat various diseases, a deeper understanding of interactive host-intestinal microbiome metabolic, signalling, and immune axes is needed^[1]^. Research on the gut microbiome has mainly focused on faecal metagenomics, while faecal metabolomics has received less attention. Metagenomics cannot detect low-molecular-weight metabolites that influence cellular metabolism and modulate phenotypes in healthy and disease states. Therefore, studying faecal low-molecular-weight metabolites is crucial for understanding host-microbial interactions in nutritional and disease research^[2-4]^. A metabolomics study of faecal samples has identified metabolites that are differentially abundant in inflammatory bowel disease (IBD)^[5]^. Additionally, a recent study analysing hundreds of faecal samples identified alterations in the metabolome of patients with IBD independent of commonly overlooked confounders such as diet and surgical history^[6]^.

Mass spectrometry (MS)-based non-targeted analysis has been employed to detect unique bacterial metabolites, for example, novel metabolomic profiles related to IBD^[5-7]^ and new gut microbiome-derived chemicals^[8]^. However, non-targeted analyses encounter major challenges related to reproducibility and quantitation, which can adversely affect data accuracy. The process of identifying metabolites often necessitates highly skilled operators, rendering it impractical for large-scale studies. Conversely, targeted metabolomics utilising triple quadrupole mass spectrometry (TQMS), which monitors both the specific precursor and product ions of each metabolite, enables precise and quantitative detection of specific metabolites^[9,10]^. This methodology is particularly advantageous for validating and screening various disease or toxicity phenotypes^[11]^. However, a major disadvantage of targeted metabolomics is its limited coverage of the metabolome^[12]^. Expanding the list of measurable metabolites in targeted metabolomics of faecal samples is crucial to studying host–intestinal microbial crosstalk.

Here, we aimed to detect as many metabolites as possible in a faecal sample by performing targeted metabolomics comprising several distinct TQMS-based methods. We strived to quantitatively identify the various metabolites from faecal samples and evaluated the potential value of the obtained data for intestinal microbiome research. This study was primarily designed as a methodological investigation to comprehensively characterise faecal metabolites. The clinical component of this study was included in an exploratory manner to demonstrate the practical applicability and analytical performance of the proposed metabolomics approach in human samples, rather than to draw definitive clinical or mechanistic conclusions. 

## METHODS

### Mice and faecal sample collection

Male germ-free (GF) mice (Jcl: MCH (ICR)/Jcl, 5-week-old) were obtained from CLEA Japan Inc. (Tokyo, Japan) and bred in separate isolators. The mice were provided water sterilised using an autoclave (121 °C, 30 min) and commercial sterilised rodent diet (CMF pellets; Oriental Yeast Co., Ltd., Japan) *ad libitum*. Isolators in which mice were bred were randomly assigned to groups: one for the GF mouse (control) group and the other for the ex-germ-free (Ex-GF) mouse (colonised) group (*n* = 8/group). Using a gastric gavage tube, 0.5 mL of 1:10 diluted fresh faeces obtained from 5-week-old conventional male Institute of Cancer Research (ICR) mice was administered to the stomach of Ex-GF mice, which were then housed until specimen collection. When the mice were 14 weeks old (9 weeks after inoculation of faecal microbiota), fresh faecal samples were individually collected, frozen immediately, and stored at -80 °C until use. Details are provided in the Supplementary Methods.

### Human volunteers and faecal sample collection

We obtained approval for this study from the Ethics Committee of Yokohama City University (YCU) Hospital (F240300037). The protocol and informed consent form received approval from the institutional ethics committee at YCU Hospital. The inclusion criteria were as follows: (1) Patients aged 20 years or older at the time of providing informed consent; (2) patients with colorectal cancer (CRC) who were undergoing endoscopic resection (ER); and (3) individuals willing to participate in the study. The exclusion criteria were as follows: (1) patients using antibiotic agents; (2) individuals using probiotics or symbiotic agents; and (3) patients judged by the investigators to be inappropriate candidates for the trial. Pre-ER faeces samples were collected 1-2 days before ER under free-living conditions without dietary restrictions and post-ER faeces samples (the first faeces within 1-2 days after ER) were obtained from five participants [Supplementary Table 1] and stored at -80 °C until analysis. Dietary intake was not formally recorded prior to sampling. Details are provided in the Supplementary Methods. The small cohort size and absence of non-CRC controls reflect the pilot nature of this analysis, which aimed solely to test the applicability of the metabolomics platform to clinical human samples.

### Preparation of metabolome from faecal samples

Faecal metabolome for various metabolites and chiral amino acids was extracted with Dulbecco’s phosphate-buffered saline (D-PBS). Faecal metabolome for short-chain fatty acids (SCFAs) was extracted with 0.1 mM 2-ethylbutyric acid in ethanol. The faecal metabolome for bile acids was extracted with 20% methanol. The faecal metabolome for phospholipid and lipid mediators was extracted with 0.1% (v/v) formic acid in methanol. Detailed faecal metabolome preparation protocols are provided in the Supplementary Methods.

### Liquid chromatography-TQMS and gas chromatography-TQMS conditions

The analytical workflows used in this study were designed to maximise metabolite coverage across diverse chemical classes rather than to achieve fully validated quantitative performance. Accordingly, the metabolomic data generated in this study should be regarded as semi-quantitative. Liquid chromatography (LC)-TQMS analysis was performed using a Nexera X3 system (Shimadzu Corporation, Kyoto, Japan) equipped with an LC-40B X3 pump, DGU-40 degasser, SIL-40C X3 autosampler, CTO-40S column oven, and CBM-40 control module. The system was coupled with an LCMS-8060 triple quadrupole mass spectrometer for mouse faecal metabolome analysis, and LCMS-8060NX for human faecal samples. Data acquisition and peak selection and integration were performed using the LabSolutions software (Shimadzu Corporation). Analysts who were blinded to the group allocation analysed the faecal metabolome.

Various metabolites, such as organic acids, nucleosides, and nucleotides, were analysed using the validated ‘LC/MS/MS (liquid chromatography coupled with tandem mass spectrometry) method package for primary metabolites version 2’ (Shimadzu Corporation). Chiral amino acids were analysed using the validated ‘LC/MS/MS method package for D- or L-amino acids (L-AAs)’ (Shimadzu Corporation). SCFAs were analysed using the validated ‘LC/MS/MS method package for short-chain fatty acids’ (Shimadzu Corporation). Bile acid analysis was conducted using the validated ‘LC/MS/MS method package bile acids version 3’ (Shimadzu Corporation), with minor modifications. Lipid mediators were analysed using the validated ‘LC/MS/MS method package for lipid mediators version 3’ (Shimadzu Corporation). Phospholipids were analysed using the validated ‘LC/MS/MS MRM library for phospholipid profiling’ (Shimadzu Corporation). GC-TQMS analysis was performed using GCMS-TQ8050 NX (a gas chromatography system coupled with a triple quadrupole mass spectrometer) with the Smart Metabolites Database (Shimadzu Corporation). Metabolite identification was performed by matching precursor and product ion *m/z* (mass-to-charge) values and retention times with those provided in the manufacturer’s validated compound libraries included in the Shimadzu method packages. Where applicable, TQMS spectral similarity was additionally considered. All metabolites that can be detected by each method package were verified by analysing each standard substance individually using each method and are listed in each link. Details of the analytical conditions and compound lists are provided in the Supplementary Methods.

### Faecal microbiota analysis using shotgun sequencing

The DNA samples were extracted from Ex-GF mouse faeces following protocol Q of Costea *et al*.^[13]^. Sequencing was performed on the MiSeq sequencer (Illumina, San Diego, CA, USA) for paired-end reads, and the adaptor sequences were trimmed. The unjoined FASTA files were analysed on the VITCOMIC2 online platform (http://vitcomic.org/), which enables genus-level taxonomical assignments by extracting and analysing nearly full-length 16S ribosomal ribonucleic acid (rRNA) gene sequences present within shotgun metagenomic data based on type strain references^[14]^. The average of total reads for each metagenome was 7.65 M (range, 5.82-9.20M) reads per sample and the average of total bases was 2.27 G (range, 1.75-2.77 G). For each faecal sample, bacterial genera below 0.1% of total detection counts were eliminated, and then percentages for the remaining genera were recalculated, which provided 46 bacterial genera across 8 samples. The ‘sample × genera’ (8 × 46) CSV (comma-separated values) table was created based on 16S rRNA gene counts in the samples.

### Data analyses

#### Unification of overlapping metabolites detected and categorisation

Metabolites identified across multiple analysis methods were unified based on the following rules. The priority for data inclusion was chiral amino acid analysis/SCFA analysis > primary metabolite analysis methods (ion-pair and pentafluorophenylpropyl [PFPP]) > GC-TQMS analysis. For GC-TQMS analysis, the handling of compounds with multiple trimethylsilyl forms was as follows:

(1) Amino acids: the compound with the largest area was selected, and the others were discarded.

(2) Sugars: compounds that were reproducible across data from eight GF mice and eight Ex-GF mice, showed the same trend between GF and Ex-GF mice, and were likely to display significant differences between the groups were retained.

For compounds overlapping in the PFPP and ion-pair methods, or for the same compounds in the GC-MS analysis, the compound with the larger area ratio was adopted. If the area ratios were comparable, selection was performed as described above for sugars. The relative area value of each metabolite among samples was normalised by dividing by the maximum value of that metabolite. All Human Metabolome Database (HMD) metabolites were classified into 44 categories based on the subclass and class of chemical taxonomy in the HMD (https://hmdb.ca/), and the universally used category names were adapted for non-listed metabolites in the HMD. 

Metabolite identification confidence was classified according to the Metabolomics Standards Initiative (MSI) guidelines. Although all metabolites included in the method packages were verified during method development using authentic reference standards, authentic standards were not analysed concurrently with the biological samples in this study. Therefore, most metabolites were classified as MSI level 2 (putatively annotated compounds). For phospholipids and carbohydrates, where structural isomers could not be fully resolved, metabolites were classified as MSI level 3 (putatively characterised compound classes).

#### Comparison of the total number of metabolites detected

The total number of metabolites detected in Ex-GF and GF mice was compared using Student’s *t*-test with GraphPad Prism 4 (GraphPad Software Inc., San Diego, CA, USA).

#### Detection of differences in relative quantities between GF and Ex-GF mice

Differences in the relative quantities of metabolites between GF and Ex-GF mice were evaluated using the Mann-Whitney U test in SPSS Statistics version 25.0 (IBM, Armonk, NY, USA). The Benjamini-Hochberg method was applied to control the false discovery rate, and *q* < 0.05 was considered statistically significant. The Benjamini-Hochberg procedure was performed using R version 3.4.4 (R Foundation for Statistical Computing, Vienna, Austria). The presence of individual metabolites in GF and Ex-GF mice was compared using Fisher’s exact test implemented in js-STAR (version XR+).

#### Spearman analysis between the meta-16S rRNA gene and metabolite concentration data

The ‘sample × metabolites’ (8 × n) CSV tables were created for 15 metabolite categories based on the concentrations of metabolites detected in the samples. Using ‘sample × genera’ (8 × 46) and ‘sample × metabolites’ (8 × n) CSV tables, Spearman analysis correlating each compound and each bacterial genus was performed on a one-to-one basis using SciPy version 1.9.1 on an Ubuntu 20.04 platform. Based on the rho scores, heatmaps with hierarchical clustering for each metabolite group on all 46 bacterial genera were created using the MetaboAnalyst 5.0 online platform (https://www.metaboanalyst.ca/MetaboAnalyst/home.xhtml). Clusterisation within metabolite groups was performed with a 60% proximity cutoff value of the dendrogram for most categories, except 55%, 70%, and 80% cutoff values were applied for D-amino acids (D-AAs), namely phosphatidylethanolamine (PtdE), bile acids, and ‘amino acids, peptides, and their analogues’, respectively. Based on this clusterisation, Spearman analysis was conducted using the arithmetic means of the concentrations of the compounds within the same cluster. This hierarchical clustering in the correlation heatmap was performed for exploratory visualisation of overall association patterns, and no permutation- or bootstrap-based statistical validation of cluster robustness was conducted. 

#### Volcano plot of metabolite concentrations between Ex-GF and GF samples

The ‘sample × metabolites’ (16 × 524) CSV table was created based on the concentrations of metabolites detected in the samples. Using the MetaboAnalyst 5.0 online platform, a volcano plot of Ex-GF samples versus GF samples was generated from this table.

#### Metabolite set enrichment analysis

Using MetaboAnalyst 5.0, metabolite set enrichment analysis (MSEA) was performed. From the 524 metabolites detected in this study, the data for 307 chemicals registered in MetaboAnalyst 5.0 were used for this analysis. Parameter Setting: The metabolite set library ‘Faeces’ was used, which contains 44 metabolite sets reported in human faeces in disease signatures.

#### Correlation network analysis

The correlation between each metabolite quantity was calculated using Spearman correlation with the Multi-omics Analysis Package run on the Garuda open platform (Shimadzu Corporation). Network graphs were generated by Cytoscape (v3.9.1) (https://cytoscape.org/) using statistically significant correlations with Pearson’s correlation coefficient |*r*| > 0.85 in specific metabolite sets detected using MSEA. Specifically, the nodes represent the metabolites investigated here and are coloured based on the Ex-GF/GF ratio of metabolite concentration or chemical category. These nodes are connected by lines (edges) showing the positive (red) and negative (blue) correlations between the analytes.

## RESULTS

### Metabolites detected using the TQMS-based multi-targeted metabolomics from mouse faecal samples

Supplementary Table 2 shows the type of metabolites detected by each analysis. LC-TQMS with ion-pair or ion-pair-free [PFPP column] analyses detected 43 and 71 metabolites, respectively, including amino acids and their derivatives, nucleosides/nucleotides, and metabolites in central metabolic pathways. GC-TQMS detected 147 metabolites, including amino acids and their derivatives, carbohydrates and their conjugates, fatty acids and their conjugates, and amines. LC-TQMS for chiral amino acid analysis detected 39 amino acids, including 19 L-AAs, 18 D-AAs, DL-proline, and glycine, which do not have a chiral centre. SCFA-targeting LC-TQMS detected 15 metabolites. Bile acid-targeting LC-TQMS detected 23 chemicals, including 12 conjugated bile acids and 11 deconjugated bile acids. Phospholipid-targeting LC-TQMS detected 201 metabolites, including phosphatidylcholine (PtdC), PtdE, phosphatidylglycerol (PtdG), phosphatidylinositol (PtdI), lysophosphatidylcholine (LPtdC), lysophosphatidylethanolamine (LPtdE), lysophosphatidylglycerol (LPtdG), lysophosphatidylserine (LPtdS), and sphingomyelin (SE). Lipid mediator-targeting LC-TQMS identified 83 metabolites, including those derived from arachidonic acid (AA), docosahexaenoic acid (DHA), and eicosapentaenoic acid (EPA). Integrating metabolites from multiple analyses yielded 524 metabolites detected using the multiplexed hybrid targeted metabolomics [Supplementary Table 3].

### Number of detected metabolites

**Figure 1:**
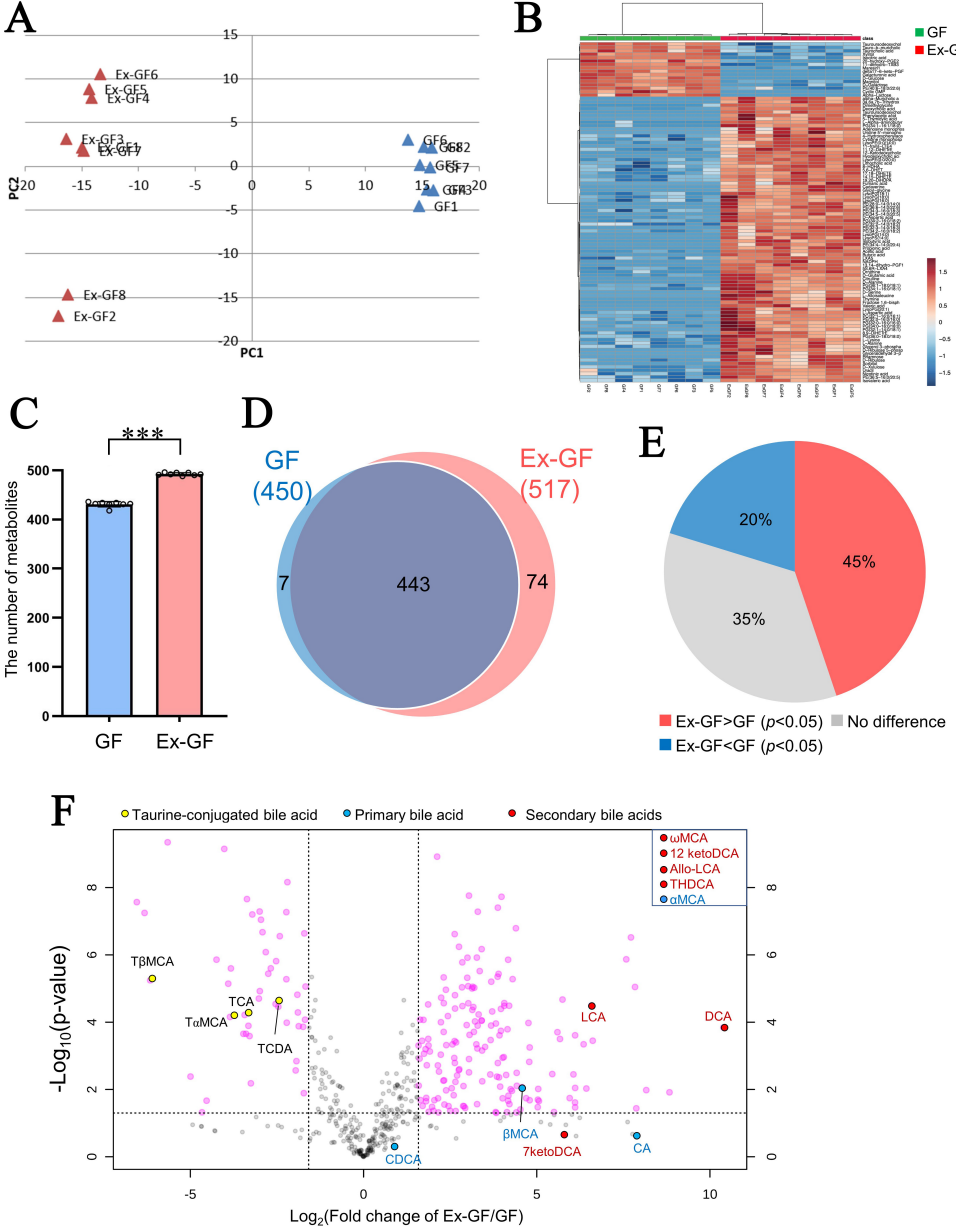
Difference between the faecal metabolomes of GF mice and Ex-GF mice. GF: Germ-free mice (*n* = 8), Ex-GF: ex-GF mice (*n* = 8). (A) Principal component analysis of metabolome profile; (B) Hierarchical clustering showing patterns of metabolites (top 100 chemicals based on *P*-value); (C) The number of metabolites detected in mouse faeces. Asterisks indicate statistical significance (***P < 0.001, Student’s *t*-test); (D) Venn diagram of faecal metabolites detected in Ex-GF and GF mice; (E) Percentage of metabolites: Ex-GF > GF, no difference, and Ex-GF < GF; (F) Volcano plot of metabolites between Ex-GF and GF mice. Taurine-conjugated bile acids and their metabolites are coloured. Metabolites in the square of the top-right are bile acids detected in only Ex-GF mice. TCA: Taurocholic acid; TCDA: taurochenodeoxycholic acid; TαMCA: tauro-α-muricholic acid; TβMCA: tauro-β-muricholic acid; CA: cholic acid; CDCA: chenodeoxycholic acid; αMCA: α-muricholic acid; βMCA: β-muricholic acid; DCA: deoxycholic acid; LCA: lithocholic acid; ωMCA: ω-muricholic acid; 7ketoDCA: 7-ketodeoxycholic acid; 12ketoDCA: 12-ketodeoxycholic acid; Allo-LCA: allolithocholic acid; THDCA: trihydroxydeoxycholic acid; GF: germ-free; Ex-GF: ex-germ-free.

The principal component analysis and hierarchical clustering results are shown in Figure 1A and B, respectively. A notable difference was observed in the faecal metabolome between GF mice and Ex-GF mice in both analyses, demonstrating that the intestinal microbiome highly influences the faecal metabolome. The total number of metabolites detected in Ex-GF mice and GF mice was 517 (the mean was 492; 488–496 metabolites/faecal sample) and 450 (the mean was 431; 418–436 metabolites/faecal sample), respectively; it was significantly higher in Ex-GF mice than in GF mice (Figure 1C, *P* < 0.001). A total of 74 and 7 out of 524 metabolites were detected in only Ex-GF mice and GF mice, respectively [Figure 1D]. Furthermore, 341 metabolites exhibited significant differences between GF and Ex-GF mice [Figure 1E], indicating that 65% of metabolites are influenced by the intestinal microbiome. Among these metabolites, the concentration of 235 metabolites was significantly higher in Ex-GF mice than in GF mice, and 106 metabolites were significantly higher in GF mice than in Ex-GF mice. A volcano plot was used to show metabolites that revealed a five-fold significant difference between Ex-GF and GF mice [Figure 1F]. By showing taurine-conjugated bile acids and their metabolites on a volcano plot, the concentrations of taurine-conjugated bile acids - taurocholic acid, taurochenodeoxycholic acid, tauro-α-muricholic acid, and tauro-β-muricholic acid - were higher in GF mice than in Ex-GF mice. The production of primary bile acids - cholic acid, chenodeoxycholic acid, α-muricholic acid, and β-muricholic acid - which are deconjugated by bacteria, was higher in Ex-GF mice. Secondary bile acids - deoxycholic acid, lithocholic acid, ω-muricholic acid, 7-ketodeoxycholic acid, 12-ketodeoxycholic acid, allolithocholic acid, and trihydroxydeoxycholic acid - which are converted from primary bile acids by bacteria, were also higher in Ex-GF mice than in GF mice. The known bacterial bile acid metabolism (i.e., the conversion of conjugated bile acids and primary bile acids to primary and secondary bile acids, respectively^[15,16]^) was observed, suggesting that intestinal bacterial metabolism can be explored using our metabolomic data.

### Categories of the detected metabolites

**Figure 2:**
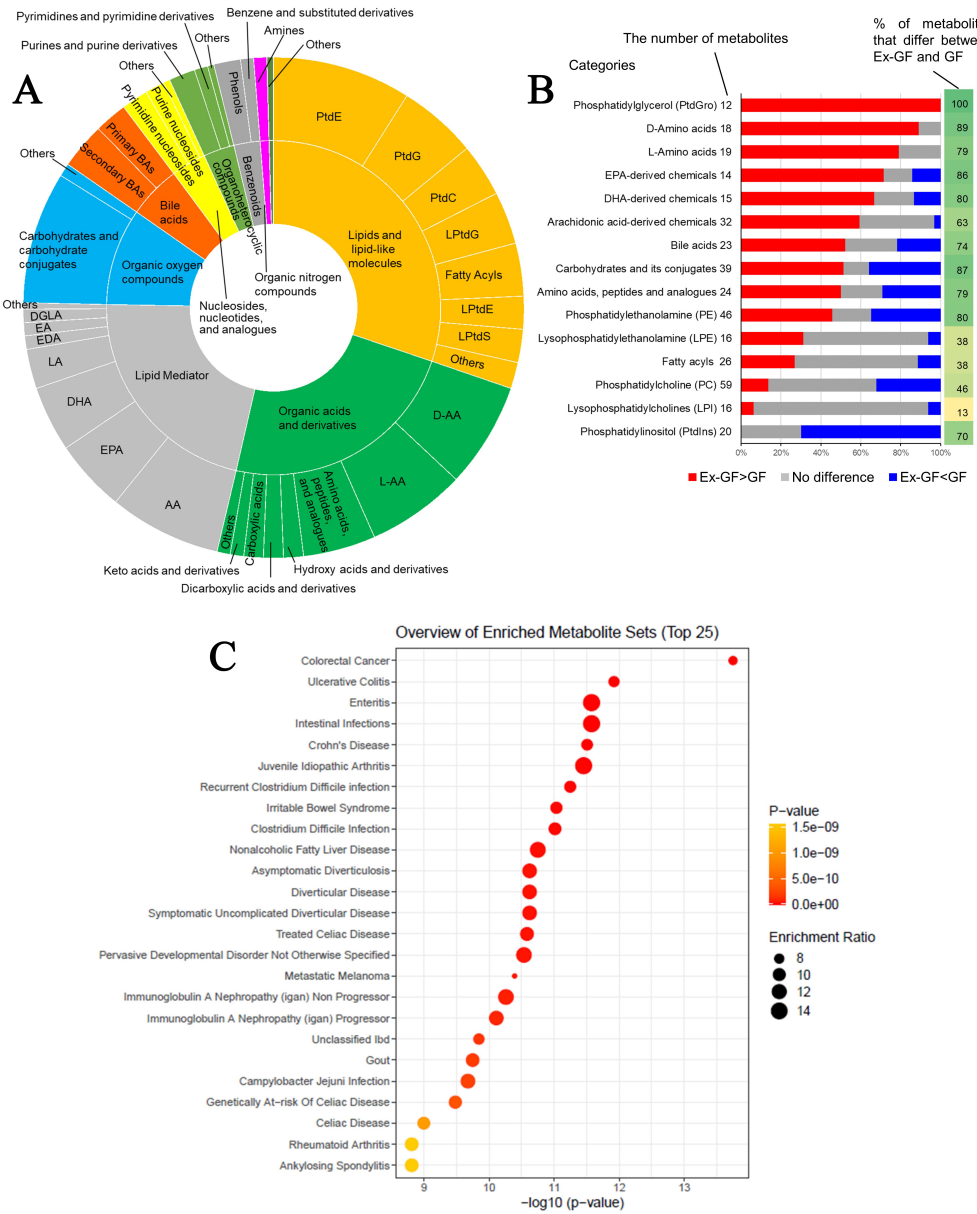
Data analyses of faecal metabolome derived from GF mice and Ex-GF mice. (A) Categories of metabolites whose concentrations were significantly higher in Ex-GF mice than in GF mice (*n* = 8/group). The number of metabolites whose concentrations were higher in Ex-GF mice than in GF mice was 235. Supplementary Figure 1 shows the categories of metabolites whose concentrations were lower in Ex-GF mice than in GF mice; (B) The proportion of metabolites increased or decreased by the intestinal microbiome in each major subclass category containing over 1% of detected metabolites. The numbers beside the category name are the number of metabolites detected by metabolomics in each category. Values on the right side of the graph are the percentage of metabolites that significantly differ between Ex-GF and GF mice; (C) Metabolite set enrichment analysis of the faecal metabolome. We identified a total of 524 metabolites, and the data of 307 of these compounds recorded in MetaboAnalyst 5.0 were utilised for analysis. For this purpose, we employed the ‘Faeces’ metabolite set library, which includes 44 metabolite sets previously documented in human faeces, to investigate disease signatures. The enrichment ratio was calculated using the formula hits/expected, where ‘hits’ refers to the observed occurrences and ‘expected’ denotes the anticipated occurrences. PtdE: Phosphatidylethanolamine; PtdG: phosphatidylglycerol; PtdC: phosphatidylcholine; PtdI: phosphatidylinositol; PtdS: phosphatidylserine; LPtdG: lysophosphatidylglycerol; LPtdE: lysophosphatidylethanolamine; LPtdS: lysophosphatidylserine; D-AA: D-amino acid; L-AA: L-amino acid; DL-AA: DL-amino acid; AA: arachidonic acid; EPA: eicosapentaenoic acid; DHA: docosahexaenoic acid; LA: Linoleic acid; EDA: eicosadienoic acid; EA: ethanolamide; DGLA: dihomo-γ-linolenic acid; GF: germ-free; Ex-GF: ex-germ-free.

All metabolites were classified into 44 categories based on the chemical taxonomy subclass and class in the HMD (https://hmdb.ca/) and commonly used categories for non-listed metabolites in the HMD [Supplementary Table 3]. Regarding metabolites whose concentrations were higher in Ex-GF mice than in GF mice, those belonging to lipids and lipid-like molecules (30.2%), organic acids and derivatives (23.4%), and lipid mediators (21.7%) comprised the majority [Figure 2A]. However, metabolites whose concentrations were lower in Ex-GF mice than in GF mice primarily included lipids and lipid-like molecules (55.6%), organic oxygen compounds (mainly carbohydrates and their conjugates) (15.1%), and organic acids and their derivatives (9.4%) [Supplementary Figure 1]. Metabolites derived from lipids and lipid-like molecules, along with organic acids and their derivatives, lipid mediators, and organic oxygen compounds, are significantly influenced by the intestinal microbiome.

Furthermore, the effect of the microbiome was analysed at the level of the chemical subclass category that contained over 1% of detected metabolites. In the categories of PtdG, D-AAs, L-AAs, EPA, DHA, bile acids, carbohydrates and their conjugates, amino acid peptides and their analogues, PtdE, and PtdI, over 70% of metabolites showed significant differences in concentrations between Ex-GF and GF mice [Figure 2B]. In particular, metabolites in the categories PtdG (100%), D-AAs (88.9%), L-AAs (78.9%), and EPA (71%) were derived from the intestinal microbiome. Notably, the concentrations of all 12 chemicals in the PtdG category were higher in Ex-GF mice than in GF mice. However, many metabolites (14/20; 70%) in PtdI were consumed by the intestinal microbiome. Although PtdG and PtdI are categorised into the same ‘lipids and lipid-like molecules’ category, the effects of the intestinal microbiome were opposite. Many lipid mediators, including pro-inflammatory mediators such as prostaglandins (PGs), thromboxanes (TXs), leukotrienes (LTs), hydroxy-eicosatetraenoic acids (HETEs), and epoxyeicosatrienoic acids (EETs), and pro-resolving lipid mediators such as resolvins, protectins, maresins, and lipoxins (LXs), were detected. Many of these were influenced by the intestinal microbiome [Supplementary Figure 2]. The analysis of metabolites in the categories LPtdE, fatty acyls, PtdC, and LPtdC revealed minimal differences in concentrations between Ex-GF and GF mice. This finding suggests that the intestinal microbiome has a limited effect on these specific metabolites.

### MSEA of the faecal metabolome

MSEA was performed to determine whether metabolites with biological or etiological significance were detected in the faecal metabolome. From the 524 metabolites detected in this study, the data for 307 metabolites were recorded in the web-based platform MetaboAnalyst 5.0 (most phospholipids and lipid mediators could not be applied), which is widely used for comprehensive metabolomics data analysis and interpretation^[17]^, and were used for this analysis. This analysis showed that several notable gastrointestinal diseases, including CRC, ulcerative colitis, enteritis, intestinal infection, Crohn's disease, and *Clostridium difficile* infection, were significantly enriched by the metabolite sets (*P* < 5 × 10^-10^; Figure 2C). 

### Relationship between the materials used, energy source, and metabolites produced by microbes in a set of metabolites related to CRC

**Figure 3:**
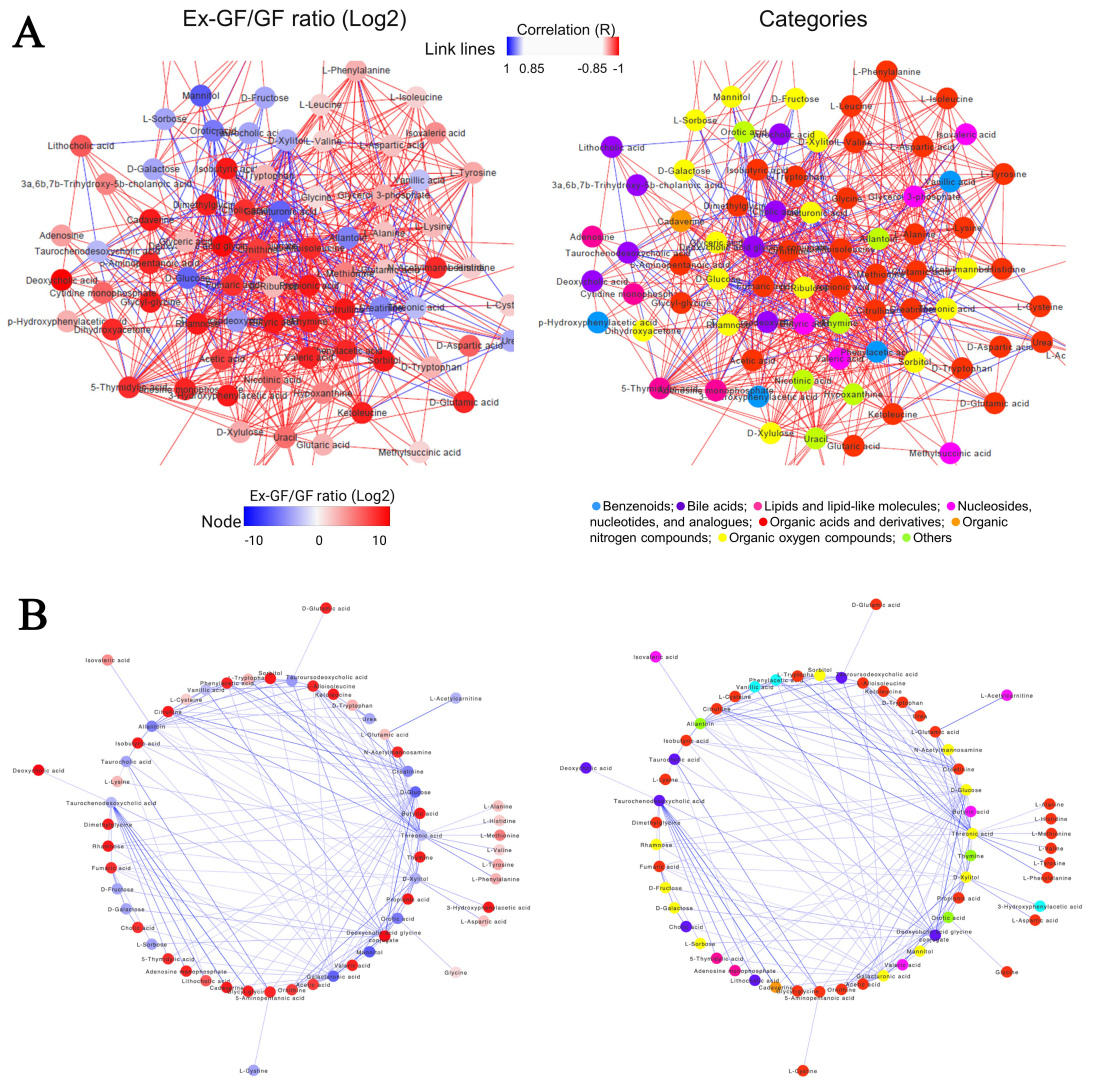
Correlation networks of significantly correlated faecal metabolites contained in the metabolite set library of colorectal cancer detected using MSEA (Spearman correlation: |*r*| > 0.85) (A) Correlation network coloured based on the ratio of Ex-GF/GF. Node colour denotes the Ex-GF/GF ratio of each metabolite (left). The correlation network is coloured based on the chemical category of metabolites (right). Node colour denotes the chemical category of each metabolite. The colour of the lines (edge) represents a positive (red) or negative (blue) correlation. The main networks were extracted from the original figures (Supplementary Figures 3 and 4 for Ex-GF/GF ratio and categories, respectively); (B) The negative correlations extracted from the upper data are based on the ratio of Ex-GF/GF (left) and the chemical category of metabolites (right). Node colours are the same as those in the upper figures. GF: Germ-free; Ex-GF: ex-germ-free; MSEA: metabolite set enrichment analysis.

We analysed the relationship among metabolites classified in the CRC-related metabolite set detected using MSEA. Figure 3A shows the result of the correlation network analysis with 100 metabolites having a Spearman correlation coefficient of > |0.85|. To understand the chemical categories of these metabolites, each metabolite was coloured according to its chemical category [Figure 3A and Supplementary Figure 3]. These metabolites were divided into eight categories, demonstrating that our TQMS-based multi-targeted metabolomics can simultaneously analyse metabolites in various chemical categories involved in CRC, and we found a correlation between these metabolites. Negative correlations may be the relationship between the substrate/precursor/energy source and the produced metabolites due to microbial metabolism. We extracted the negative correlations from this data and analysed them [Figure 3B and Supplementary Figure 4]. D-Glucose, threonic acid, taurochenodeoxycholic acid, D-xylitol, galacturonic acid, creatinine, and allantoin were negatively associated with over six metabolites, whose concentrations were higher in Ex-GF than in GF mice.

### Integrated analysis of 16S rRNA gene sequences and metabolome data

**Figure 4:**
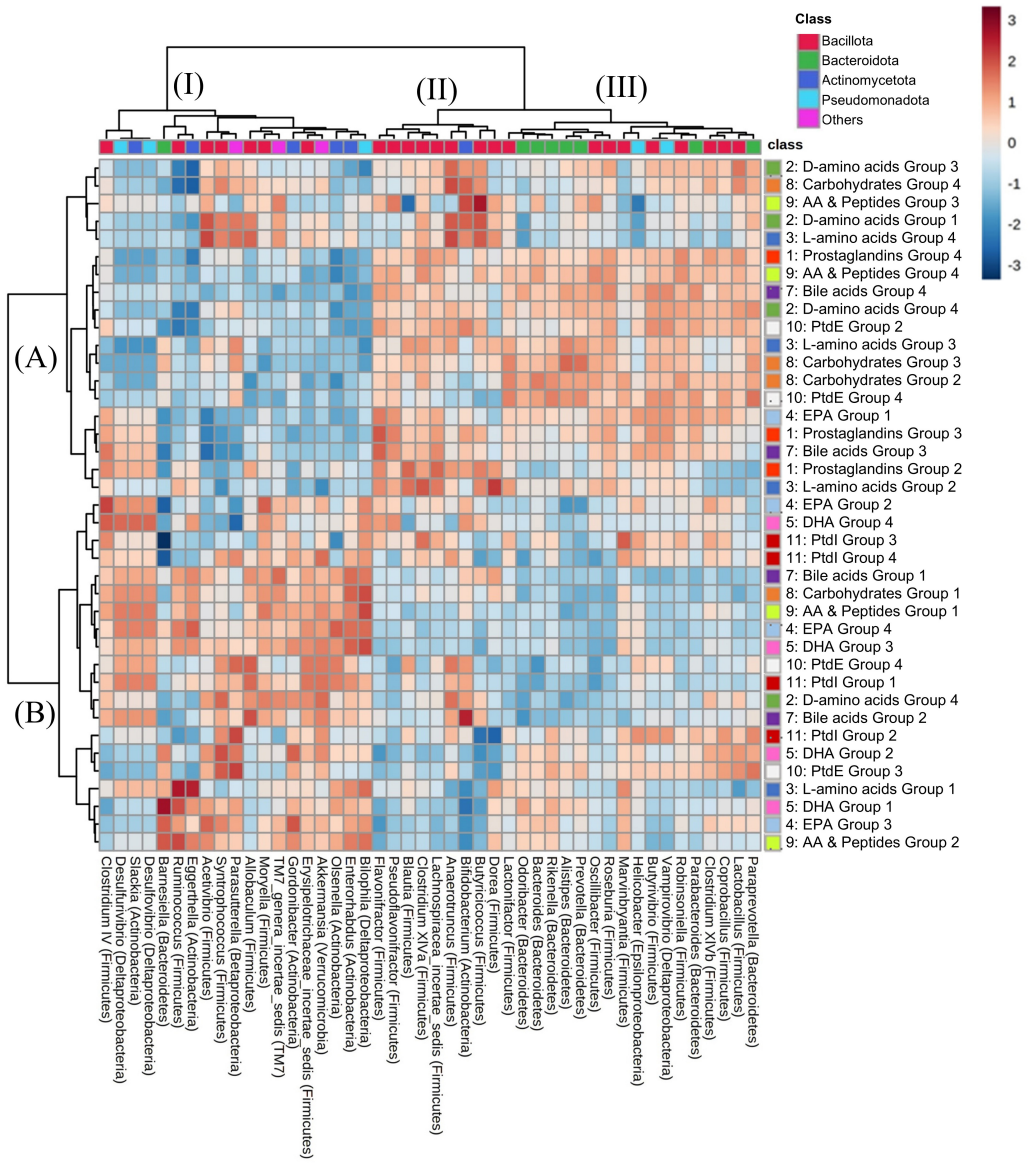
Correlation analysis of bacterial composition and metabolome. A heatmap representing Spearman’s rank correlation between the abundance of the metabolite group and the relative abundance of the genus based on the 16S rRNA gene sequence. Supplementary Figures 5-14 show the metabolites in groups. AA: Arachidonic acid; PtdE: phosphatidylethanolamine; EPA: eicosapentaenoic acid; DHA: docosahexaenoic acid; PtdI: phosphatidylinositol.

To investigate the relationship between the microbiome and metabolome in faecal samples, an integrated analysis was performed, combining metabolomics with genus-level microbiome profiles derived from 16S rRNA gene sequences extracted from shotgun metagenomic data. As taxonomic ranks lower than the family level are preferable for 16S rRNA gene-based analysis to investigate the correlation between the microbiome and metabolome^[18]^, genus-level analyses were performed in this study. Spearman’s rank correlation between faecal bacterial profiles and metabolite profiles between Ex-GF and GF mice was analysed, where bacterial genera of 0.1% or more among all reads and metabolomes in faecal samples were mapped. To simplify hierarchical clustering and heatmap interpretation, we analysed the correlation between bacterial composition and the metabolome in each category and divided metabolome profiles into three to four groups per category based on the hierarchical clustering pattern and threshold [Supplementary Figures 5-14]. Next, we analysed the correlation between the bacterial profile and metabolome using classified groups in all metabolome categories influenced by the intestinal microbiome [Figure 4]. Hierarchical clustering separated the bacterial profiles into three groups: Bacillota (Firmicutes)-enriched (II), mainly Bacillota (Firmicutes) and Bacteroidota (Bacteroidetes) (III), and mixed bacterial classes (including Bacillota, Bacteroidota, Actinomycota, and Pseudomonadota) (I). The profile of the metabolites was roughly divided into two clusters (A and B in Figure 4). Notably, except for DHA and PtdI, metabolite groups were not classified per the chemical categories.

### Metabolites detected using the TQMS-based multi-targeted metabolomics from clinical faecal samples

**Figure 5:**
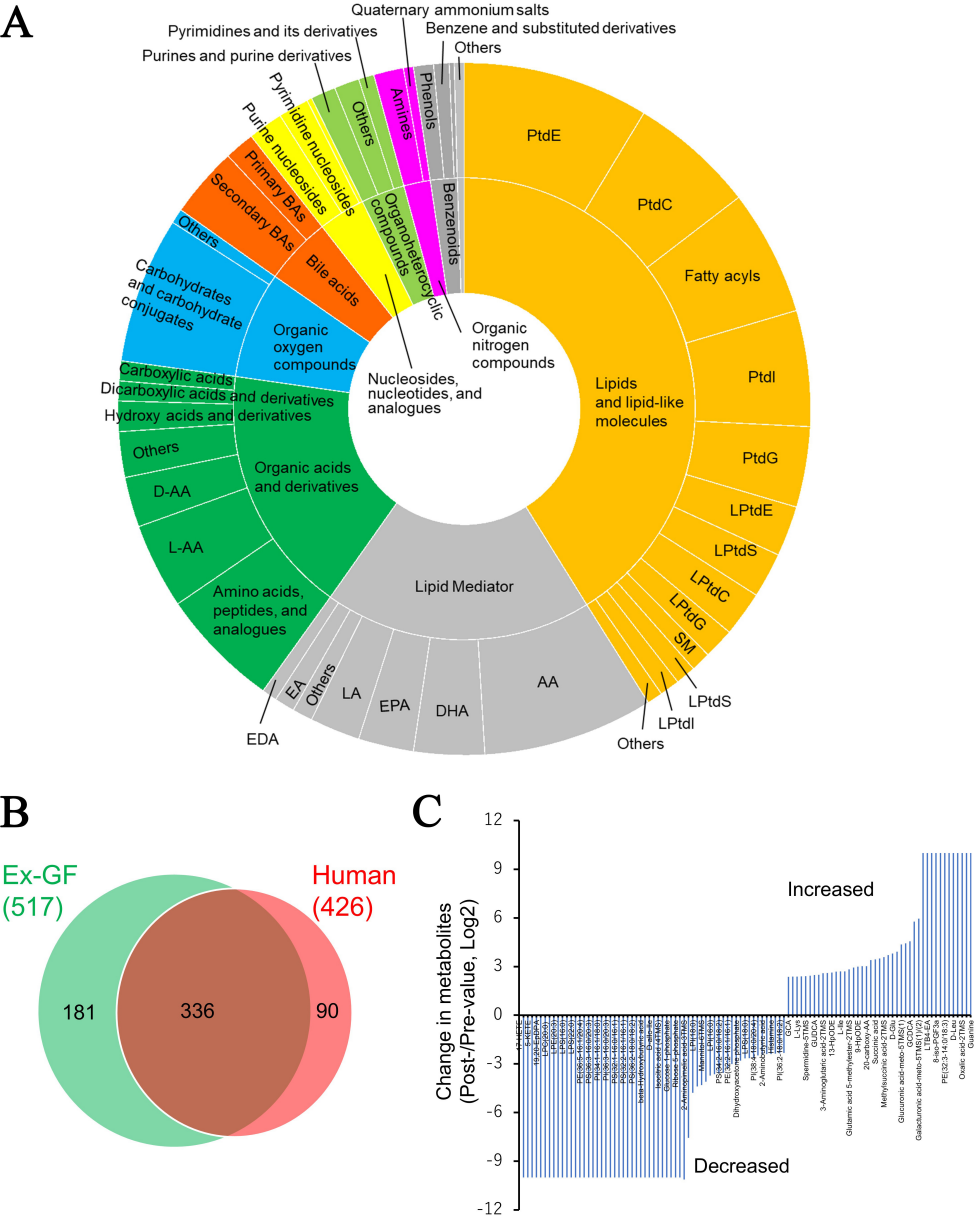
Faecal metabolome derived from human patients with colorectal cancer (*n* = 5). (A) Categories of the metabolites detected in human faeces. For definitions of the abbreviated metabolite names refer to the legend of Figure 2; (B) Venn diagram of faecal metabolites detected in Ex-GF mice and humans; (C) Detection of metabolites altered by endoscopic resection in patient No. 1. Metabolites whose concentrations increased or decreased by more than five-fold before and after endoscopic treatment are shown. Values for metabolites that were undetected before treatment but detected after treatment, and for metabolites detected before treatment but undetected after treatment, were assigned 10 and -10, respectively. Supplementary Table 6 shows the detailed metabolites and levels of change of all metabolites, including unchanged metabolites. PtdE: Phosphatidylethanolamine; PtdC: phosphatidylcholine; PtdI: phosphatidylinositol; PtdG: phosphatidylglycerol; LPtdE: lysophosphatidylethanolamine; PtdS: phosphatidylserine; LPtdC: lysophosphatidylcholine; LPtdG: lysophosphatidylglycerol; SM: sphingomyelin; LPtdS: lysophosphatidylserine; LPtdC: lysophosphatidylcholine; LPtdI: lysophosphatidylinositol; AA: arachidonic acid; DHA: docosahexaenoic acid; EPA: eicosapentaenoic acid; LA: Linoleic acid; EA: ethanolamide; EDA: eicosadienoic acid; D-AA: D-amino acid; L-AA: L-amino acid.

To assess the relevance of our metabolomics platform for clinical applications, we conducted an initial analysis of the faecal metabolome from five patients with CRC. This investigation was prompted by findings based on metabolic profiling in murine models, which revealed that intestinal microbiome-derived metabolites are linked to various gastrointestinal diseases, including CRC [Figure 2C]. A total of 426 metabolites were detected in the faecal metabolome derived from patients with CRC (mean: 357; 309–389 types of metabolites/faecal sample). These metabolites were classified based on the chemical taxonomy, and Figure 5A shows the percentages of each category. Metabolites belonging to lipids and lipid-like molecules (47.0%), lipid mediators (18.8%), and organic acids and derivatives (17.4%) comprised the majority. Overall, 607 metabolites were detected in the faecal metabolome of both humans and mice [Supplementary Table 4]. The metabolome in the faeces of humans and Ex-GF mice was compared. A total of 90 out of 426 metabolites and 181 out of 524 metabolites were detected in only human and Ex-GF mice, respectively [Figure 5B]. MSEA, using data from 241 metabolites registered in the web-based platform MetaboAnalyst 6.0, demonstrated enrichment of CRC-related metabolite sets and showcased the platform’s potential for identifying clinically relevant metabolic signatures in human faecal samples (Holm *P* = 7.77e-15). Other gastrointestinal diseases were also significantly enriched by metabolite sets [Supplementary Table 5]. Faecal samples were collected from one patient both before and after ER of CRC, and the changes in metabolome were analysed. This metabolomic analysis revealed a large alteration of the faecal metabolome: the concentrations of 62 and 43 metabolites out of 386 metabolites were increased and decreased by more than 5-fold, respectively, by ER [Figure 5C and Supplementary Table 6]. However, as dietary intake was not controlled and the observation was based on a very small cohort, these findings should be interpreted with caution. Nevertheless, our approach highlights the feasibility of monitoring faecal metabolome dynamics in clinical studies. It is ideal to assess changes in gut bacteria-related substances associated with clinical interventions in controlled clinical trials.

## DISCUSSION

To enhance the repertoire of measurable low-molecular-weight metabolites for studies on the gut microbiome, this research was conducted using TQMS-based multi-targeted metabolomics. This method offers high sensitivity, specificity, and robust quantification^[19]^. We successfully identified a remarkable total of 607 distinct metabolites in faecal samples. Additionally, various targeted metabolomics approaches were employed to analyse specific chemical categories, including SCFAs, L- and D-AAs, and bile acids^[20-24]^. Previous wide-targeted metabolomic studies have identified approximately 70-220 types of metabolites in human or mouse faecal samples, and this number is relatively small^[25-29]^. Importantly, our multi-targeted metabolomic analysis detected metabolites in various chemical categories, including chiral amino acids, bile acids, SCFAs, carbohydrates, nucleic acids, amines, and lipids, such as phospholipids and lipid mediators. Previously, wide-targeted metabolomics using LC^[25,27,29]^ and capillary electrophoresis^[26,28]^ had been used for faecal analyses. The use of GC in our analysis significantly enhanced metabolite detection, particularly in the carbohydrate category, which is not typically identified through LC or capillary electrophoresis methods. Additionally, data on lipids, including phospholipids and lipid mediators, also contributed to the increase in the number of metabolites detected in this analysis. To our knowledge, there is just one report on faecal metabolome analysis using multi-targeted metabolomics with GC-MS and LC-MS, annotating 298 metabolites^[30]^. The number of metabolites detected in this study is the highest ever detected in a targeted metabolomics study in both human and mouse faecal samples. 

We have updated the list of metabolites influenced by the intestinal microbiome by comparing the faecal metabolomes derived from GF and Ex-GF mice based on semi-quantitative data. The identification of 235 metabolites whose concentrations were significantly higher in Ex-GF than in GF mice can provide new insights. Furthermore, the MSEA revealed that gastrointestinal disease-related metabolite sets were enriched in the faecal metabolome in both humans and mice, demonstrating that our targeted metabolomics enables comprehensive profiling of disease-annotated metabolites in faecal samples. Through additional correlation network analyses targeting the CRC-annotated metabolite set, known bacterial metabolic relationships, such as the precursor-product associations previously reported in the context of CRC studies, were observed. For example, an association consistent with the reported bacterial conversion of taurochenodeoxycholic acid to the secondary bile acid lithocholic acid was observed. Moreover, there was a negative correlation between several carbohydrates, such as D-glucose and D-xylitol, and several chemicals, such as propionate. This association is consistent with that observed in a previous report study, that is, intestinal microbes can metabolise dietary carbohydrates into propionate^[31]^. Overall, our multi-targeted metabolomics approach enables detection of relationships among metabolites, even when they belong to different categories. However, it should be emphasised that the enrichment of CRC-annotated metabolites in Ex-GF mice does not indicate disease phenotypes or pathological processes; rather, it reflects microbial contributions to metabolite production previously reported in CRC studies. While this study focused on semi-quantitative metabolomics, the methodology can be adapted for relative or absolute quantification when appropriate authentic standards are available.

Our multi-targeted metabolomics also provides new insights into the effects of the intestinal microbiome on lipid metabolites, including lipid mediators and phospholipids. Lipid mediators have recently been the subject of concern owing to their bioactive functions, and several reports have shed light on bioactive lipid chemicals produced by the intestinal microbiome^[32]^. Specifically, 10-hydroxy-cis-12-octadecenoic acid, produced by intestinal bacteria from linoleic acid^[33,34]^, has several functions; for example, it can ameliorate dextran sodium sulphate-induced colitis^[35]^. Polyunsaturated fatty acid (PUFA)-derived substances are recognised as key factors in immune regulation and disease control. Particularly, the metabolic balance between ω-6 and ω-3 PUFAs is vital for health^[36-38]^. ω-6 PUFAs are conventionally involved in initiating inflammatory responses. In the body, AA, metabolised from linoleic acid classified as ω-6 PUFAs, is converted into pro-inflammatory mediators, such as PGs, LTs, HETEs, and EETs. As shown in Figure 3B, several PGs, LTs, and HETEs were significantly higher in Ex-GF mice than in GF mice, indicating that the intestinal microbiome is associated with multiple pro-inflammatory lipid mediators, considered endogenous, in faecal samples. In the case of human faeces, 6 PGs and 11 HETEs were detected, indicating that the human gut microbiome also produces pro-inflammatory lipid mediators. In contrast, ω-3 PUFAs seem to promote inflammation resolution. α-Linolenic acid, classified as an ω-3 PUFA, is converted into precursors for EPA and DHA in the body. Specialised pro-resolving lipid mediators, including resolvins, protectins, and maresins, which ameliorate inflammation-sustaining processes such as angiogenesis, pro-inflammatory cytokine release, and the clearance of apoptotic cells and microorganisms, are synthesised in the body from essential EPA or DHA^[38,39]^. Although LXs are mainly converted from ω-6 PUFA-related AA, they work as pro-resolving lipid mediators^[40]^. In this study, we found that the levels of two LXs (5S,6R-LXA4 and LXA5) were significantly higher in Ex-GF mice than in GF mice, suggesting a potential link between the intestinal microbiome and lipid mediators previously implicated in inflammation resolution. These findings may help develop new medical strategies. In contrast, the levels of DHA-derived resolving D2 and maresin 1 (pro-resolving lipid mediators) and TXs (TXB1 and 11-dehydro-TXB3; pro-inflammatory lipid mediators) were significantly lower in Ex-GF mice than in GF mice. This suggests that these lipid mediators may be released into the colonic lumen by colonocytes but are catabolised or absorbed by the intestinal microbiome. The lipid mediator precursors, including AA, EPA, and DHA, are also produced by the intestinal microbiome, and these are crucial to producing lipid mediators by the intestinal microbiome. This finding suggests that the bacterial composition may be associated with shifts in the balance of lipid mediators, namely pro-resolution and pro-inflammation, in the colonic lumen. Pro-inflammatory HETEs show strong positive correlations with *Olsenella*, *Enterorhabdus*, *Eggerthella*, and *Slackia* in the Actinomycetota (Actinobacteria) phylum; *Desulfovibrio*, *Desulfurivibrio*, and *Bilophila* in the Pseudomonadota (Deltaproteobacteria) phylum; and *Ruminococcus* in the Bacillota (Firmicutes) phylum. Previous studies have shown that bacteria in Pseudomonadota (Deltaproteobacteria) are likely to be involved in inflammation in the disease model. For example, the intestinal *Desulfovibrio* level increased with inflammation level in rotenone-induced Parkinson’s disease model mice^[41]^ and increased along stages of non-alcoholic fatty liver disease-associated hepatocellular carcinoma formation^[42]^. Further studies are required to confirm that these lipid mediators are transported to the host cells from the colonic lumen. In this study, although correlation analyses between microbial taxa and metabolites were performed in mice, similar integrated analyses in human samples were not conducted because of the small cohort size and the lack of appropriate controls. Such integrative analyses of well-characterised human cohorts will be essential to strengthen the translational relevance of future studies.

This study focused on comparisons among simultaneously measured sample groups and did not compare different batches or animal species. However, as internal standards are added to each method, comparisons of different batches of samples or cross-platform comparisons are also possible. Our metabolomics technology can semi-quantify various metabolites in human and mouse faeces, including chiral amino acids, carbohydrates, SCFAs, bile acids, lipid mediators, and phospholipids. This is the first study in which such a wide range of chemicals was detected in these samples using targeted metabolomics. Many of the metabolites that we identified are associated with gastrointestinal diseases. We observed patterns consistent with microbial involvement in the presence of these metabolites. This technology can assist in clinical trials aimed at discovering new bioactive metabolites in the faecal sample.

This study has some limitations. First, the human CRC cohort was very small (*n* = 5) and lacked non-CRC controls, making the human study strictly proof-of-concept analysis. In addition, the human and murine datasets were not intended for direct biological comparison; ex-GF mice samples were included solely to demonstrate methodological applicability across experimental systems rather than disease-related relevance. Second, the metabolomic data were not normalised by faecal dry weight, total ion current (TIC), or internal biomarkers. Dry-weight normalisation was not feasible because no additional faecal material was available. As faecal water content was not measured, differences in faecal hydration may have influenced relative metabolite abundance. Therefore, potential effects of faecal water content on fold-change and correlation analyses cannot be fully excluded, and the results should be interpreted with caution. TIC-based normalisation was not applied because this study combined multiple targeted TQMS-based methods with distinct analytical characteristics, making the TIC values not directly comparable across methods. In addition, there are no widely accepted endogenous biomarkers for neutral normalisation in faecal metabolomics. Therefore, the metabolite concentrations should be interpreted with caution, especially when comparing values across methods. Third, formal quality control procedures, such as pooled quality control sample analysis, peak area stability assessment, and drift correction, were not implemented. As a result, potential batch effects related to instrument performance or analytical runs cannot be fully excluded. Regarding the analytical performance characteristics, limited validation metrics (e.g., linearity ranges, coefficient of determination (R^2^) values, and within-day reproducibility) were available for selected classes such as bile acids, SCFAs, lipid mediators, phospholipids, and chiral amino acids; linearity ranges and R² values were available for PFPP-based and ion-pair analyses. However, comprehensive validation parameters, including limit of detection/limit of quantitation, between-day reproducibility, matrix effects in faecal samples, and recovery efficiencies, were not assessed for any of the analytical methods [Supplementary Table 7]. Therefore, this multi-targeted metabolomics approach should be interpreted as semi-quantitative, and analytical bias cannot be fully excluded. Fourth, dietary intake prior to pre-ER sampling was not standardised or recorded, which may have contributed to inter-sample variability in the faecal metabolome. Although post-ER samples were collected during hospitalisation under standardised dietary conditions, dietary effects cannot be completely excluded. Fifth, functional validation of disease-associated metabolites was not performed. Sixth, although predefined prioritisation rules were applied, cross-platform reproducibility metrics were not formally assessed; hence, potential platform-dependent biases cannot be fully excluded. In addition, the unification of overlapping metabolites across multiple assays remains challenging and requires further software development. Finally, the field needs to increase the analysable metabolites; for example, isoallolithocholic acid (bile acid), which contributes to longevity^[43]^, and 10-hydroxy-cis-12-octadecenoic acid, which has several functions^[35]^, are off-target in this metabolomic database. These limitations highlight that our primary aim was to establish and demonstrate the workflow, rather than to draw definitive disease-related conclusions.

In conclusion, we developed a TQMS-based multi-targeted metabolomics workflow that enables the relative quantification of over 600 faecal metabolites across diverse chemical categories. Using GF and Ex-GF mice, we demonstrated the utility of the workflow in defining microbiota-dependent metabolites, including bile acid transformations and lipid mediator production. A proof-of-concept analysis of faeces of patients with CRC illustrated the feasibility of extending this workflow to analysing human clinical samples. However, the small cohort size precludes any disease-specific conclusions. Therefore, our principal contribution is methodological: providing a broadly applicable, reproducible platform that can serve both basic microbiome research and future translational studies. Studies in larger and more diverse clinical cohorts will be required to validate disease associations suggested by this platform.

## DECLARATIONS

### Availability of data and materials

The faecal microbiota analysis data have been deposited in the DNA Data Bank of Japan (DDBJ) Sequence Read Archive under accession numbers DRR683203-DRR683210. Raw metabolomics data were deposited in MB-POST [Raw metabolomics data were deposited in the MB-POST (https://repository.massbank.jp/), a metabolomics mass spectrometry data repository, under accession numbers MPST000174, MPST000177, MPST000167, MPST000173, MPST000172, MPST000180 and MPST000181]. Other data supporting the findings of this study are available in the supplementary tables.

### Copyright

© The Author(s) 2026.
