# Supplementary material for: Development of a multi-targeted metabolomics platform for semi-quantification of faecal metabolites: a proof-of-concept analysis in human faeces
Source: Microbiome Res Rep. 2026 Mar 26;5(1):7. doi: 10.20517/mrr.2025.85 (PMC13092893; doi:10.20517/mrr.2025.85)
Supplement: Supplementary file 1 [file mrr-5-1-7-SupplementaryMaterials.zip › mrr4085-SupplementaryMaterials.pdf]

Microbiome Research Reports

Supplementary materials

Development of a multi-targeted metabolomics platform for semi-quantification of faecal metabolites: a proof-of-concept analysis in human faeces

Kayo Ikuta[1#], Akihiro Kunisawa[2,3#], Itaru Dekio[4#], Arisa Ito[3], Qiuyi Wang[2,3], Kazuhiro Kawamura[2,3,6], Masaki Yamada[2], Sumi Nakamura[1,7], Yoshihiro Hayakawa[2], Takuma Higurashi[5], Junko Iida[2,3], Eiichiro Fukusaki[3,6], Toru Suzuki[8,9], Mitsuharu Matsumoto[1,3]

[1]Dairy Science and Technology Institute, Kyodo Milk Industry Co. Ltd, Tokyo 190-0182, Japan.

[2]Shimadzu Corporation, Kyoto 604-8511, Japan.

[3]The University of Osaka and Shimadzu Analytical Innovation Research Laboratories, The University of Osaka, Osaka 565-0871, Japan.

[4]Department of Dermatology, The Jikei University School of Medicine, Minato-ku, Tokyo 105-8461, Japan.

[5]Department of Gastroenterology and Hepatology, Yokohama City University School of Medicine, Yokohama 236-0004, Japan.

[6]Graduate School of Engineering, The University of Osaka, Osaka, 565-0871, Japan

[7]BC LAB, Osaka 541-0047, Japan.

[8]Department of Cardiovascular Sciences, University of Leicester and NIHR Leicester Biomedical Research Centre, Leicester LE1 7RH, UK.

[9]The Institute of Medical Science, The University of Tokyo, Tokyo 108-0071, Japan.

[#]These authors contributed equally to this work.

Correspondence to: Dr. Mitsuharu Matsumoto, Research Laboratories, Kyodo Milk Industry Co. Ltd, Tokyo 190-0182, Japan. E-mail: m-matumoto@metio.co.jp

ORCID: Mitsuharu Matsumoto (0000-0002-0378-3077)

Supplementary Methods: page 2–7

Supplementary Figure 1. Categories of metabolites that were significantly lower in

Ex-GF mice than in GF mice (n = 8/group).

Supplementary Figure 2. Difference in the representative lipid mediators between GF mice and Ex-GF mice

Supplementary Figure 3. Correlation networks of significantly correlated faecal metabolites contained in metabolite set library of colorectal cancer detected by MSEA (Spearman correlation: r > |0.85|)

Supplementary Figure 4. Correlation networks of significantly correlated faecal metabolites contained in metabolite set library of colorectal cancer detected by MSEA (Spearman correlation: r > |0.85|).

Supplementary Figure 5. Correlation analysis of bacterial composition and metabolome focusing phosphatidylglycerol (PG)

Supplementary Figure 6. Correlation analysis of bacterial composition and metabolome focusing D-amino acids.

Supplementary Figure 7. Correlation analysis of bacterial composition and metabolome focusing L-amino acids.

Supplementary Figure 8. Correlation analysis of bacterial composition and metabolome focusing eicosapentaenoic acid (EPA)

Supplementary Figure 9. Correlation analysis of bacterial composition and metabolome focusing docosahexaenoic acid (DHA).

Supplementary Figure 10. Correlation analysis of bacterial composition and metabolome focusing bile acids.

Supplementary Figure 11. Correlation analysis of bacterial composition and metabolome focusing carbohydrate and its conjugates.

Supplementary Figure 12. Correlation analysis of bacterial composition and metabolome focusing amino acids (excluding basic L- and D-amino acids), peptides and its analogues

Supplementary Figure 13. Correlation analysis of bacterial composition and metabolome focusing phosphatidylethanolamine (PE).

Supplementary Figure 14. Correlation analysis of bacterial composition and metabolome focusing phosphatidylinositol (PI).

Supplementary Table 1–7 are provided as Excel file.

METHODS

Mice and faecal sample collection

Two sterile boxes of 5-week-old male germ-free (GF) mice (Jcl:MCH (ICR)/Jcl) (8 mice in each box) were obtained from CLEA Japan Inc. (Tokyo, Japan) and bred in separate isolators at the Research Laboratories of Kyodo Milk Industry Co. Ltd. Isolators in which mice are bred were randomly assigned to groups: one for the GF mice (control) group and the other for the ex-GF mice (colonized) group (n = 8/group). The isolator sterilisation methodology, water and CMF pellets provision (Oriental Yeast Co. Ltd., Tokyo, Japan), and faecal microbiota inoculation were adapted from a previous report[1]. In brief, mice were provided water sterilised using an autoclave (121 °C, 30 min) and commercial sterilised CMF pellets *ad libitum*. Using a gastric gavage tube, 0.5 mL of 1:10 diluted fresh faeces obtained from 5-week-old conventional male ICR mice was administered to the stomach of Ex-GF mice, which were then housed until specimen collection. At 14 weeks old (9 weeks after faecal microbiota inoculation), fresh faecal samples were individually collected, frozen immediately, and stored at -80 °C until use. Animal experiments were approved by the Kyodo Milk Animal Use Committee (permit number: 2019-37) and followed the Guide for the Care and Use of Laboratory Animals, published by the National Academies Press.

Human volunteers and faecal sample collection

Study coordination, registration and data collection were conducted in the Department of Gastroenterology and Hepatology at Yokohama City University (YCU) Hospital. The study protocol complied with the Declaration of Helsinki and the Ethics Guidelines for Clinical Research published by the Ministry of Health, Labor and Welfare, Japan. We obtained approval for this study from the Ethics Committee of YCU Hospital on May 1, 2024 (F240300037). The protocol and informed consent form received approval from the institutional ethics committee at YCU Hospital. The inclusion criteria were as follows: 1) aged 20 years or older on the date of informed consent; 2) patients with CRC who were undergoing endoscopic resection (ER); 3) willingness to participate in the study. The exclusion criteria were as follows: 1) use of antibiotic agents; 2) use of probiotics or symbiotic agents; 3) patients judged by the investigators to be inappropriate candidates for the trial. Pre-ER faeces samples were collected 1–2 days before ER under free-living conditions without dietary restrictions and post-ER faeces samples (the first faeces within 1–2 days after ER) were obtained from five participants

(Supplementary Table 1). All faecal samples collected were stored at -80 °C until analysis.

Preparation of metabolome from faecal samples

Analysts who were unaware of the group allocation prepared the faecal metabolome. We used LC-TQMS to identify various metabolites using ion-pair and ion-pair-free (pentafluorophenylpropyl column) analyses and GC-TQMS to identify diverse metabolites. For LC-TQMS using ion-pair and ion pair-free analyses, faecal samples (approximately 50 mg) were diluted 10-fold with GIBCO® Dulbecco's phosphate buffered saline (D-PBS) (Thermo Fisher Scientific, Waltham, MA, USA) containing DL-Alanine-d4 (50 μM) as an internal standard and extracted twice by subjecting the sample to intense mixing for 1 min and incubating for 5 min on an icebox without agitation. After the extraction, the upper aqueous portion without big precipitation at the bottom was collected and centrifuged (10,000 ×*g* for 10 min at 4 °C), and 200 μL of the supernatant was centrifugally filtered using Amicon Ultra Centrifugal Filters, 3 kDa (Merck, Darmstadt, Germany) for mouse faeces and 5-kDa cut-off filter Ultrafree-MC-PLHCC (MilliporeSigma, Burlington, MA, USA) for human faeces. The filtrate was stored at -80 °C until further use. Before the LC-TQMS analyses, 5 μL of the filtrate was added to 1 μM MES in 45 μL of the mobile phase, comprising a mixture of 15 mM acetic acid per 10 mM tributylamine-water for ion-pair analysis and a mobile phase comprising a mixture of 0.1% (v/v) formic acid per water for ion-pair-free analysis. For GC-TQMS, faecal samples (approximately 50 mg) were diluted 10-fold with water extraction, centrifugation, and cut-off filtration were performed as described for LC-TQMS using ion-pair and ion-pair-free analyses. The filtrate was diluted 10-fold with water, followed by the addition of 10 μL of 2-isopropylmalic acid (0.5 mg/mL) as an internal standard solution and 250 μL of methanol-chloroform-water (2.5:1:1), mixed for 1 min, and incubated for 30 min at 37 °C with shaking. A total of 225 μL of the supernatant was collected, to which 200 μL of water was added, followed by mixing for 1 min and centrifugation (16,000 ×*g* for 3 min at 4 °C). A total of 250 μL of the supernatant was collected and dried using a vacuum evaporator. The dried samples were dissolved in 20 mg/mL methoxyamine in 80 μL of pyridine, followed by ultrasonication (20 min), and incubated for 90 min at 30 °C with shaking. Following this, 40 μL of N-Methyl-N-trimethylsilyltrifluoroacetamide was added to the solutions

and incubated for 30 min at 37 °C with shaking. The derivatised solutions were centrifuged (16,000 ×*g* for 3 min at 25 °C), and the supernatant was collected. To analyse chiral amino acids using LC-TQMS, the faecal metabolome was prepared with the LC-TQMS method using ion-pair and ion-pair-free analyses. Before LC-TQMS analysis, 30 μL of the filtrate was dried using a vacuum evaporator (Eppendorf Concentrator plus) and dissolved in a mobile phase comprising a mixture of 80% acetonitrile, 15% ethanol, 5% water, and 0.5% trifluoroacetic acid (all v/v).

To analyse SCFAs using LC-TQMS, faecal samples (approximately 8 mg) were diluted 37.5-fold with 0.1 mM 2-ethylbutyric acid (Sigma-Aldrich, St. Louis, MO, USA) in ethanol as an internal standard solution, extracted by intense mixing for 1 min and incubated for 5 min on an icebox. The extracts were centrifuged (16,000 ×*g* for 10 min at 4 °C), and 50 μL of the supernatant was collected in a 1.5 mL tube. Three reagents, namely, 50 mM 3-Nitrophenylhydrazine hydrochloride (Sigma-Aldrich), 50 mM 1-ethyl-3-(3-dimethylaminopropyl)carbodiimide hydrochloride (Sigma-Aldrich), and 7.5% (v/v) pyridine, were prepared for the derivatisation reaction. All reagents were dissolved in 75:25 methanol:water (v/v). Next, 50 μL of each of these three solutions was added to the extracts and the solution was mixed with a shaker for 30 min at approximately 25 °C for a derivatisation reaction. The derivatised solutions were diluted five-fold with 0.5% (v/v) formic acid in 75% (v/v) methanol to stop derivatisation.

For bile acid analysis, faecal samples (20 mg) were dissolved in 80 μL of water and 20 μL of 100 μM internal standard in methanol. The internal standard solution comprised 10 types of stable isotopes, namely [$^{2}H_4$]-chenodeoxycholic acid, [$^{2}H_4$]-cholic acid, [$^{2}H_4$]-deoxycholic acid, [$^{2}H_4$]-glycocholic acid, [$^{2}H_4$]-glycodeoxycholic acid, [$^{2}H_4$]-glycolithocholic acid, [$^{2}H_4$]-lithocholic acid, [$^{2}H_4$]-taurochenodeoxycholic acid, [$^{2}H_4$]-taurocholic acid, and [$^{2}H_4$]-taurolithocholic acid (Alsachim, Illkirch-Graffenstaden, France). A total of 30 μL of 1 mol/L hydrochloric acid and 1,000 μL of acetonitrile were added to the faecal solutions and extracted twice by intense mixing for 1 min and incubating for 5 min on an icebox. The extracts were centrifuged (14,000 ×*g* for 15 min at 25 °C), and 500 μL of the supernatant was collected in a 1.5-mL tube and evaporated to dryness with a vacuum concentrator (2,000 rpm, 120 min). The dried samples were

dissolved in 100 μL of methanol and mixed by shaking (1 min) and ultrasonication (10 min). The solutions were centrifuged (14,000 ×*g* for 15 min at approximately 25 °C), and the supernatants were diluted 100-fold with methanol.

For phospholipid and lipid mediators detection, faecal samples (approximately 20 mg) were dissolved in 500 μL of 0.1% (v/v) formic acid/methanol and 20 μL of the internal standard mixture solution (50 ppb tetranor-PGEM-d6, 6-keto-PGF1α-d4, TXB2-d4, PGF2α-d4, PGE2-d4, PGD2-d4, LTC4-d5, LTD4-d5, PGA2-d4, LTB4-d4, 14,15-DiHET-d11, 15-HETE-d8, 12-HETE-d8, 5-HETE-d8, PAF-d4, 11,12-EET-d11, OEA-d4, and AA-d8 / methanol). After being crushed with a bead shocker (MicroSmash Ms-100, TOMY, Tokyo, Japan) (5,000 rpm for 1 min), the extract was centrifuged (13,000 ×*g* for 15 min at 4 °C), and 400 μL of the supernatant was collected in a 1.5-mL tube. We used 50 μL of the extract for phospholipid analysis. The remaining solution was subjected to the following pretreatment for lipid mediator analysis. Next, 1,400 μL of 0.1% formic acid/water solution was added to the extract solution and the solution was loaded onto a solid-phase extraction column (Strata-X, 10 mg, Phenomenex, Torrance, CA, USA) pre-conditioned with 1 mL of 0.1% formic acid/methanol and 1 mL of 0.1% formic acid. The column was washed with 1 mL of 0.1% formic acid, followed by 1 mL of a 0.1% formic acid/15% ethanol/water solution, and then 1 mL of hexane, in that order. To elute the lipids, a 0.1% formic acid/methanol solution was added. Following evaporation, the eluents were redissolved in methanol. All water used for the metabolome preparation was ultrapure water.

Liquid chromatography-triple-quadrupole mass spectrometry conditions

LC-MS/MS analysis was performed using a Nexera X3 system (Shimadzu Corporation, Kyoto, Japan) for mouse faecal metabolome or LCMS-8060NX (Shimadzu Corporation) for human faecal metabolome equipped with an LC-40B X3 pump, a DGU-40 degasser, an SIL-40C X3 autosampler, a CTO-40S column oven, and a CBM-40 control module, coupled with an LCMS-8060 triple quadrupole mass spectrometer (Shimadzu Corporation). Data acquisition, peak selection, and integration were performed using the LabSolutions software (Shimadzu Corporation). Analysts who were unaware of the group allocation analysed the faecal metabolome.

Primary metabolites such as organic acids, nucleosides, and nucleotides were analysed using the validated 'LC/MS/MS method package for primary metabolites version 2' (Shimadzu Corporation) (https://www.shimadzu.eu/products/liquid-chromatograph-mass-spectrometry/lc-ms-method-packages/primary-metabolites/index.html). The Discovery HS F5-3 column (2.1 mm internal diameter × 150 mm length, 3-μm particle size; Sigma-Aldrich) was used (PFPP method). The mobile phase comprised a mixture of 0.1% (v/v) formic acid water and 0.1% (v/v) formic acid-acetonitrile. The flow rate, column temperature, and injection volume were set at 0.25 mL/min, 40 °C, and 3 μL, respectively. The mass spectrometer was equipped with an electrospray ionisation (ESI) source under the following conditions: nebulising gas flow, 3 L/min; heating gas flow, 10 L/min; interface temperature, 300 °C (Nexera X3 system)/270 °C (LCMS-8060NX system); desolvation line temperature, 250 °C; heating block temperature, 400 °C; and drying gas flow, 10 L/min. The collision-induced dissociation gas pressure was set to 270 kPa. The relative area (peak area value) of each compound was calculated to the internal standard 2-morpholinoethanesulfonic acid. To analyse more polar metabolites, such as sugar phosphates, the Mastro C18 column for the mouse faecal metabolome or the Mastro2 C18 for the human faecal metabolome (2.0 mm internal diameter × 150 mm length, 3-μm particle size; Shimadzu GLC) was used (ion-pair method). The mobile phase comprised a mixture of 15 mM acetic acid per 10 mM tributylamine-water and methanol. The flow rate, column temperature, and injection volume were set at 0.3 mL/min, 40 °C, and 3 μL, respectively. The mass spectrometer was equipped with an ESI source under the following conditions: nebulising gas flow, 2 L/min; heating gas flow, 10 L/min; interface temperature, 300 °C (Nexera X3 system)/270 °C (LCMS-8060NX system); desolvation line temperature, 250 °C; heating block temperature, 400 °C; and drying gas flow, 10 L/min. The collision-induced dissociation gas pressure was set to 270 kPa. The relative area (peak area value) of each compound was calculated to that of the internal standard 2-morpholinoethanesulfonic acid.
Chiral amino acids were analysed using the validated 'LC/MS/MS method package for D/L amino acids' (Shimadzu Corporation) (https://www.shimadzu.eu/products/liquid-chromatograph-mass-spectrometry/lc-ms-method-packages/dl-amino-acids/index.html) based on a previous report[2].

SCFAs were analysed using the validated 'LC/MS/MS method package for short-chain fatty acids' (Shimadzu Corporation) (https://www.shimadzu.com/an/products/liquid-chromatograph-mass-spectrometry/lc-ms-system/lcmsms-method-package-for-short-chain-fatty-acids/index.html?utm_source=chatgpt.com). The Mastro C18 column was used for mouse faeces, whereas the Mastro2 C18 column was used for human faeces (2.0 mm internal diameter × 150 mm length, 3-μm particle size; Shimadzu GLC). The mobile phase comprised a mixture of 0.1% (v/v) formic acid, water, and acetonitrile. The flow rate, column temperature, and injection volume were set at 0.35 mL/min, 40 °C, and 3 μL, respectively. The mass spectrometer was equipped with an ESI source under the following conditions: nebulising gas flow, 2 L/min; heating gas flow, 10 L/min; interface temperature, 300 °C (Nexera X3 system)/270 °C (LCMS-8060NX system); desolvation line temperature, 250 °C; heating block temperature, 400 °C; and drying gas flow, 10 L/min. The collision-induced dissociation gas pressure was set to 270 kPa. The relative area (peak area value) of each compound was calculated to that of the internal standard 2-ethylbutyric acid.

Bile acid analysis was conducted using the validated 'LC/MS/MS method package bile acids ver. 3' (Shimadzu Corporation) (https://www.shimadzu.eu/products/liquid-chromatograph-mass-spectrometry/lc-ms-method-packages/bile-acids/index.html), with minor modifications. The Inertsil ODS-4 HP column (2.1 mm internal diameter × 150 mm length, 3-μm particle size; GL Sciences) was used. The mobile phase comprised a mixture of 0.05% (v/v) acetic acid, water, and 50% (v/v) acetonitrile/methanol. The flow rate, column temperature, and injection volume were set at 0.3 mL/min, 30 °C, and 2 μL, respectively. The mass spectrometer was equipped with an ESI source under the following conditions: nebulising gas flow, 2 L/min; heating gas flow, 10 L/min; interface temperature, 300 °C (Nexera X3 system)/270 °C (LCMS-8060NX system); desolvation line temperature, 250 °C; heating block temperature, 400 °C; and drying gas flow, 10 L/min. The collision-induced dissociation gas pressure was set to 190 kPa. The relative area, represented by the peak area value, of each compound was determined using ten internal standards of their stable isotope, specifically 2,2,4,4-d4. Lipid mediators were analysed using the validated 'LC/MS/MS method package for lipid mediators ver.3' (Shimadzu Corporation) (https://www.shimadzu.eu/products/liquid-chromatograph-mass-spectrometry/lc-ms-

method-packages/lipid-mediators/index.html) based on that of Yamada *et al.*[3]. The Kinetex C18 column (2.1 mm internal diameter × 150 mm length, 2.6-μm particle size; Phenomenex, Torrance, CA) was used. The mobile phase comprised a mixture of 0.1% (v/v) formic acid, water, and acetonitrile. The flow rate, column temperature, and injection volume were set at 0.4 mL/min, 40 °C, and 5 μL, respectively. The mass spectrometer was equipped with an ESI source under the following conditions: nebulising gas flow, 2.5 L/min; heating gas flow, 10 L/min; interface temperature, 270 °C; desolvation line temperature, 250 °C; heating block temperature, 400 °C; and drying gas flow, 10 L/min. The collision-induced dissociation gas pressure was set to 230 kPa. The relative area (peak area value) of each compound was calculated to those of 18 internal standards of their stable isotope.

Phospholipids were analysed using the validated 'LC/MS/MS MRM library for phospholipid profiling' (Shimadzu Corporation) (https://www.shimadzu.com/an/products/liquid-chromatograph-mass-spectrometry/lc-ms-software/lcmsms-mrm-library-for-phospholipid-profiling/index.html). The Kinetex C18 column (2.1 mm internal diameter × 150 mm length, 2.6-μm particle size; Phenomenex) was used. The mobile phase comprised a mixture of 20 mM ammonium formate-water and 50% (v/v) acetonitrile / 2-propanol. The flow rate, column temperature, and injection volume were set at 0.3 mL/min, 45 °C, and 3 μL, respectively. The mass spectrometer was equipped with an ESI source under the following conditions: nebulising gas flow, 2 L/min; heating gas flow, 10 L/min; interface temperature, 300 °C; desolvation line temperature, 250 °C; heating block temperature, 400 °C; and drying gas flow, 10 L/min. The collision-induced dissociation gas pressure was set to 230 kPa.

Gas chromatography-triple-quadrupole mass spectrometry conditions

GC-TQMS analysis was performed using GC-MS-TQ8050 NX with Smart Metabolites Database (Shimadzu Corporation) (https://www.shimadzu.com/an/products/gas-chromatograph-mass-spectrometry/gc-ms-software/smart-metabolites-database/index.html). A fused silica capillary column BPX-5 (30 m × 0.25 mm inner diameter; film thickness: 0.25 μm) (Trajan Scientific and Medical, Melbourne, Australia) was used. Analysis conditions were as follows: constant flow rate of helium,

39.0 cm/s; ion source temperature, 200 °C; electron ionisation, 70 eV; injection volume, 1 μL; injection, pulsed split (split ratio, 1:30); and oven temperature, 60 °C for 2 min, increased at a rate of 15 °C/min to 330 °C and maintained at that temperature for 3 min. Data acquisition, peak selection, and integration were conducted using the LabSolutions Insight software (Shimadzu Corporation).

16S rRNA gene-based meta-analysis

The DNA samples were extracted from Ex-GF murine faeces by protocol Q of Costea *et al.*[4]. Sequencing was performed on the MiSeq sequencer (Illumina, San Diego, CA, USA) for paired-end reads, and the adaptor sequences were trimmed. The unjoined fasta files were analysed on the VITCOMIC2 online platform (http://vitcomic.org/), which enables genus-level taxonomical assignments by extracting and analysing nearly full-length 16S rRNA gene sequences present within shotgun metagenomic data based on type strain references[5]. For each faecal sample, bacterial genera below 0.1% of total detection counts were eliminated, and then percentages for the remaining genera were recalculated, which provided 46 bacterial genera across 8 samples. The 'sample × genera' (8 × 46) CSV table was created based on 16S rRNA gene counts in the samples.

REFERENCES

1. Matsumoto M, Kunisawa A, Hattori T, et al. Free D-amino acids produced by commensal bacteria in the colonic lumen. *Sci Rep* 2018;8:17915.10.1038/s41598-018-36244-z.
2. Nakano Y, Konya Y, Taniguchi M, Fukusaki E. Development of a liquid chromatography-tandem mass spectrometry method for quantitative analysis of trace d-amino acids. *J Biosci Bioeng* 2017;123:134-8.10.1016/j.jbiosc.2016.07.008.
3. Yamada M, Kita Y, Kohira T, et al. A comprehensive quantification method for eicosanoids and related compounds by using liquid chromatography/mass spectrometry with high speed continuous ionization polarity switching. *J Chromatogr B Analyt Technol Biomed Life Sci* 2015;995-996:74-84.10.1016/j.jchromb.2015.05.015.
4. Costea PI, Zeller G, Sunagawa S, et al. Towards standards for human fecal sample processing in metagenomic studies. *Nat Biotechnol* 2017;35:1069-76.10.1038/nbt.3960.
5. Mori H, Maruyama T, Yano M, Yamada T, Kurokawa K. VITCOMIC2: visualization tool for the phylogenetic composition of microbial communities based on

16S rRNA gene amplicons and metagenomic shotgun sequencing. *BMC Syst Biol* 2018;12:30.10.1186/s12918-018-0545-2.

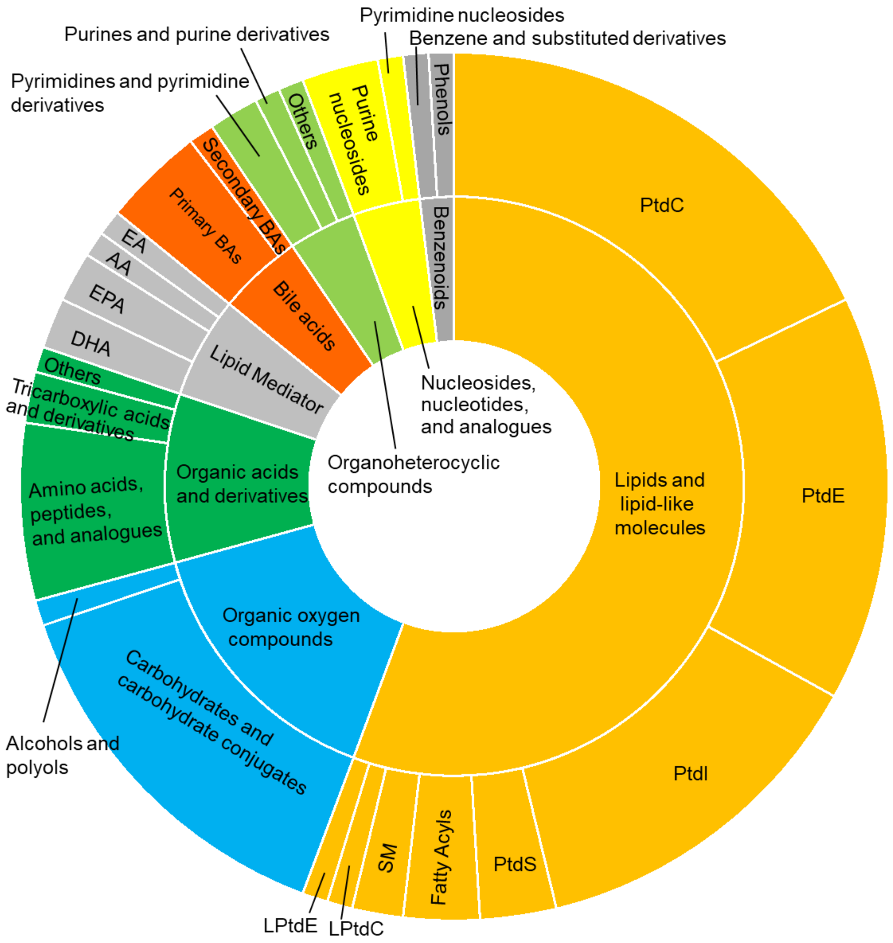

Supplementary Figure 1. Categories of metabolites that were significantly lower in Ex-GF mice than in GF mice (n = 8/group). The number of metabolites that were lower in Ex-GF mice than in GF mice was 106. Categories of metabolites that were higher in Ex-GF mice than in GF mice are shown in Figure 2A.

PtdE: Phosphatidylethanolamine, PtdG: Phosphatidylglycerol, PtdC: Phosphatidylcholine, PtdI: Phosphatidylinositol, PtdS: Phosphatidylserine, LPtdG: Lysophosphatidylglycerol, LPtdE: Lysophosphatidylethanolamine, LPtdS: Lysophosphatidylserine, LPtdC: Lysophosphatidylcholine, SM: Sphingomyelin, D-AA: D-Amino acids, L-AA: L-Amino acids, DL-AA: DL-Amino acids, AA: Arachidonic acids, EPA: Eicosapentaenoic acids, DHA: Docosahexaenoic acids, LA: linoleic acids, EDA: Eicosadienoic acids, EA: Ethanolamide, DGLA: Dihomo-γ-linolenic acids

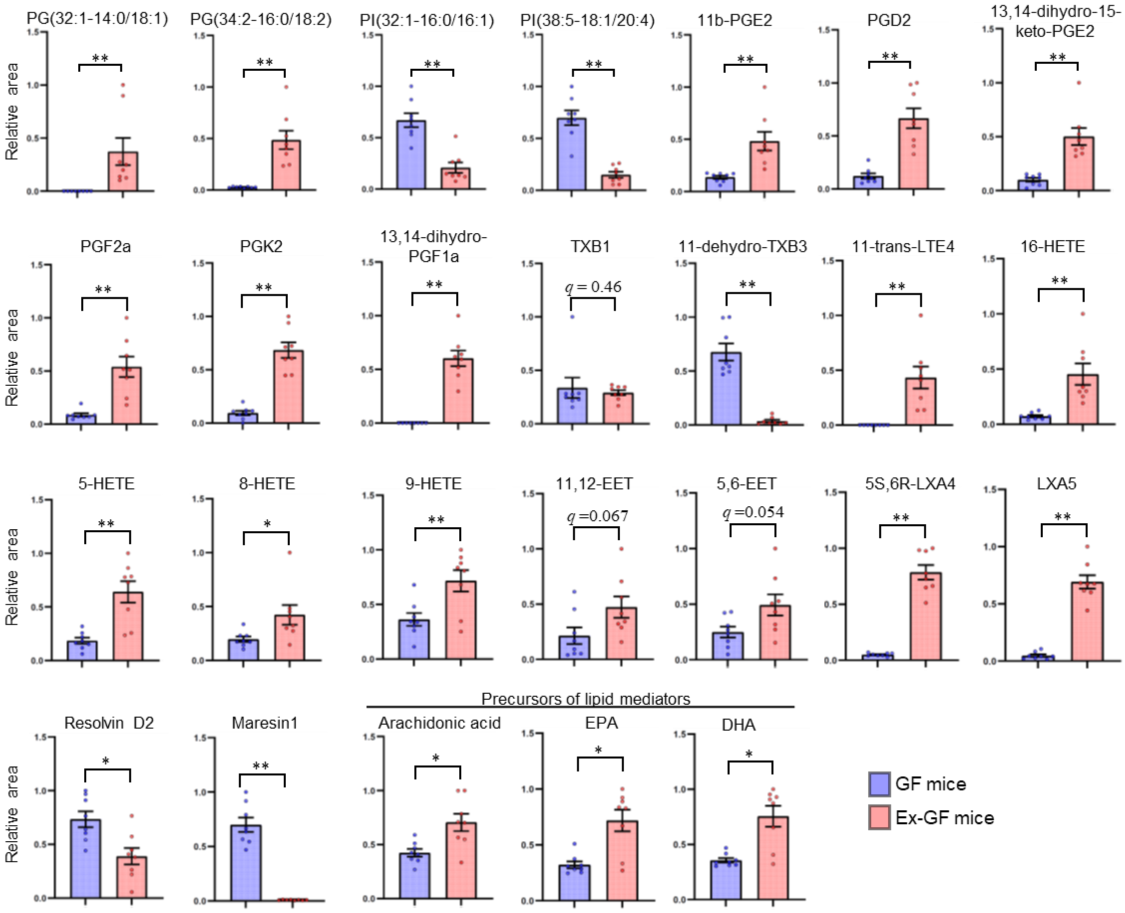

Supplementary Figure 2. Difference in the representative lipid mediators between GF mice and Ex-GF mice

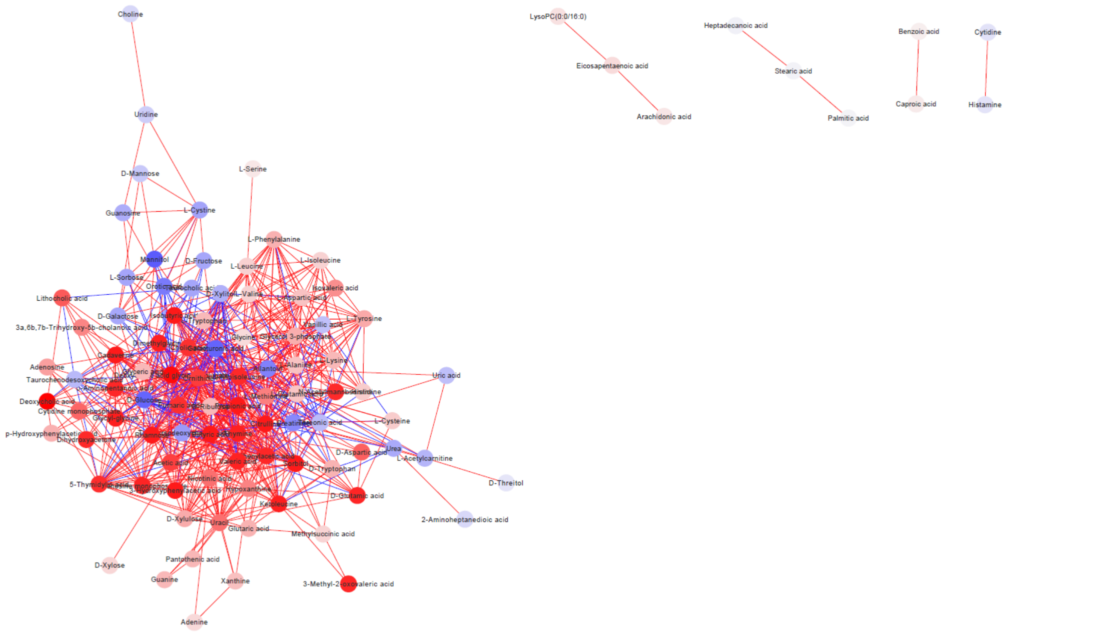

Supplementary Figure 3. Correlation networks of significantly correlated faecal metabolites contained in metabolite set library of colorectal cancer detected by MSEA (Spearman correlation: r > |0.85|). Correlation network coloured based on the ratio of Ex-GF/GF. Node colour denotes the Ex-GF/GF ratio of each metabolite. The colour of the lines (edge) represents positive (red) or negative (blue) correlation. The squared areas are extracted in Figure 3A (right).

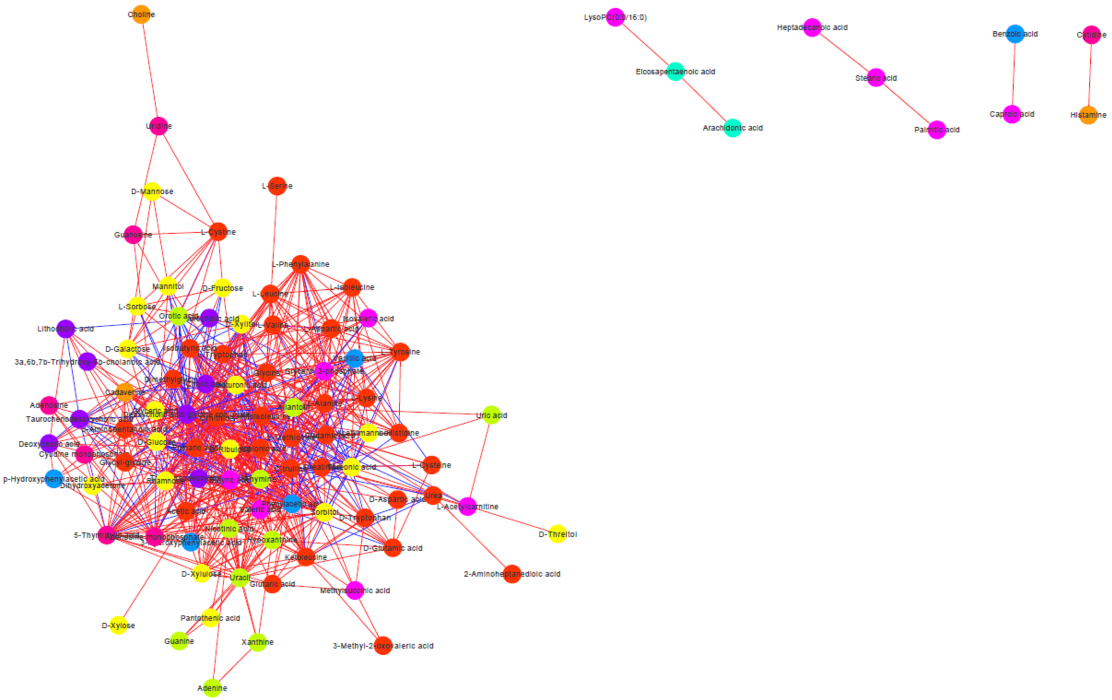

Supplementary Figure 4. Correlation networks of significantly correlated faecal metabolites contained in metabolite set library of colorectal cancer detected by MSEA (Spearman correlation: r > |0.85|). The correlation network is coloured based on the chemical category of metabolites. Node colour denotes the chemical category of each metabolite. The colour of the lines (edge) represents positive (red) or negative (blue) correlation.

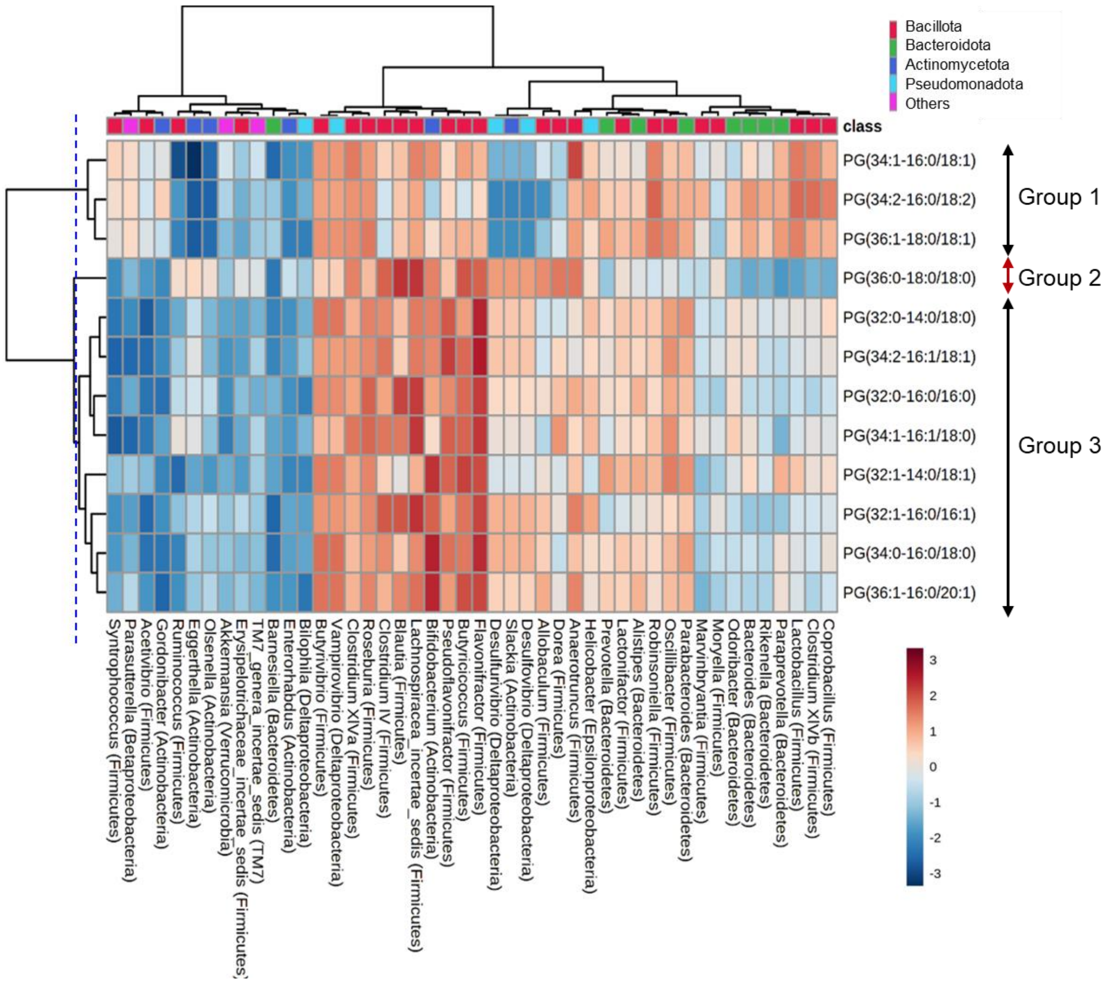

Supplementary Figure 5. Correlation analysis of bacterial composition and metabolome focusing phosphatidylglycerol (PG). Chemicals were classified into 3 groups based on threshold shown in blue dot line.

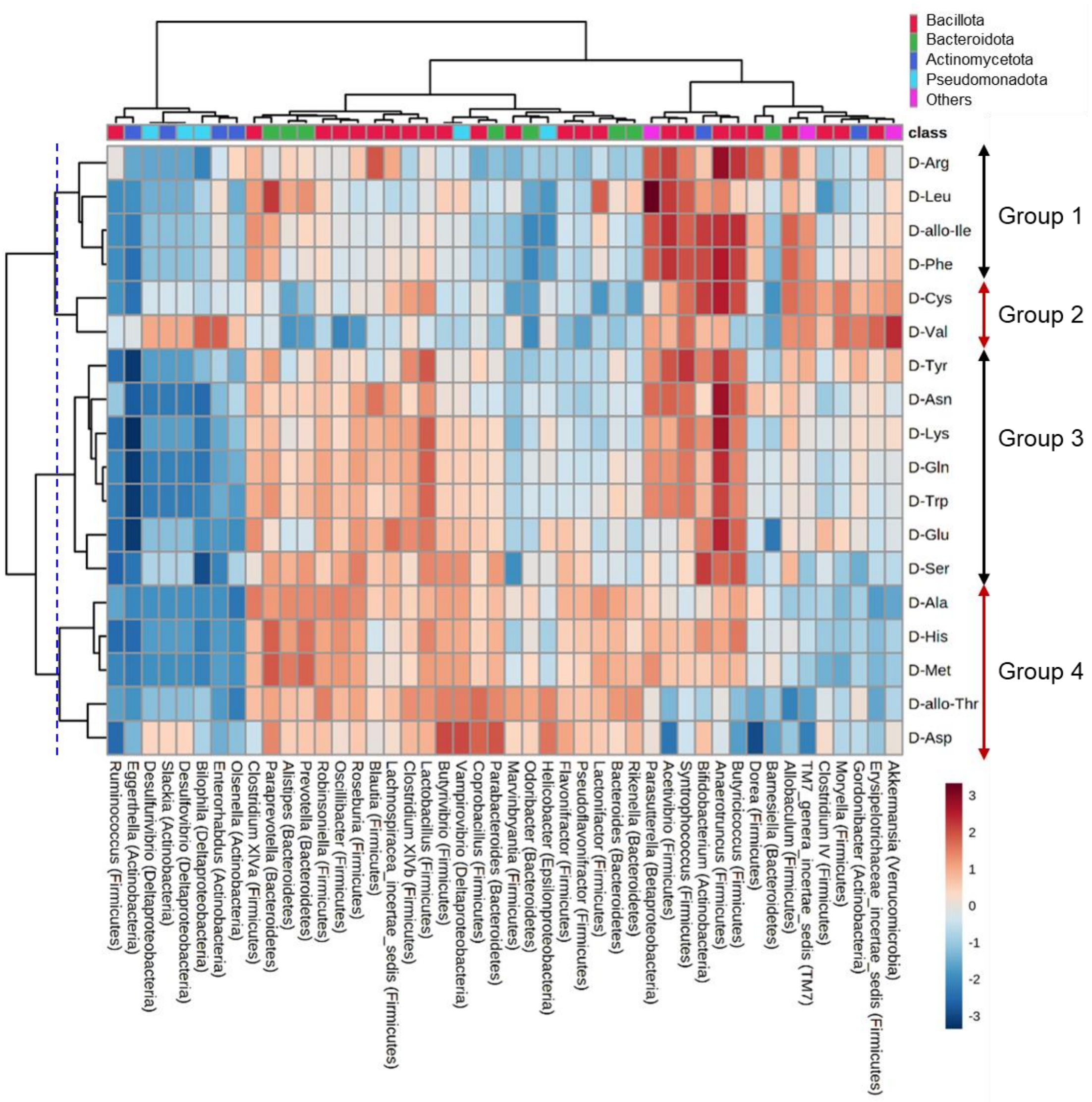

Supplementary Figure 6. Correlation analysis of bacterial composition and metabolome focusing D-amino acids. Chemicals were classified into 4 groups based on threshold shown in blue dot line.

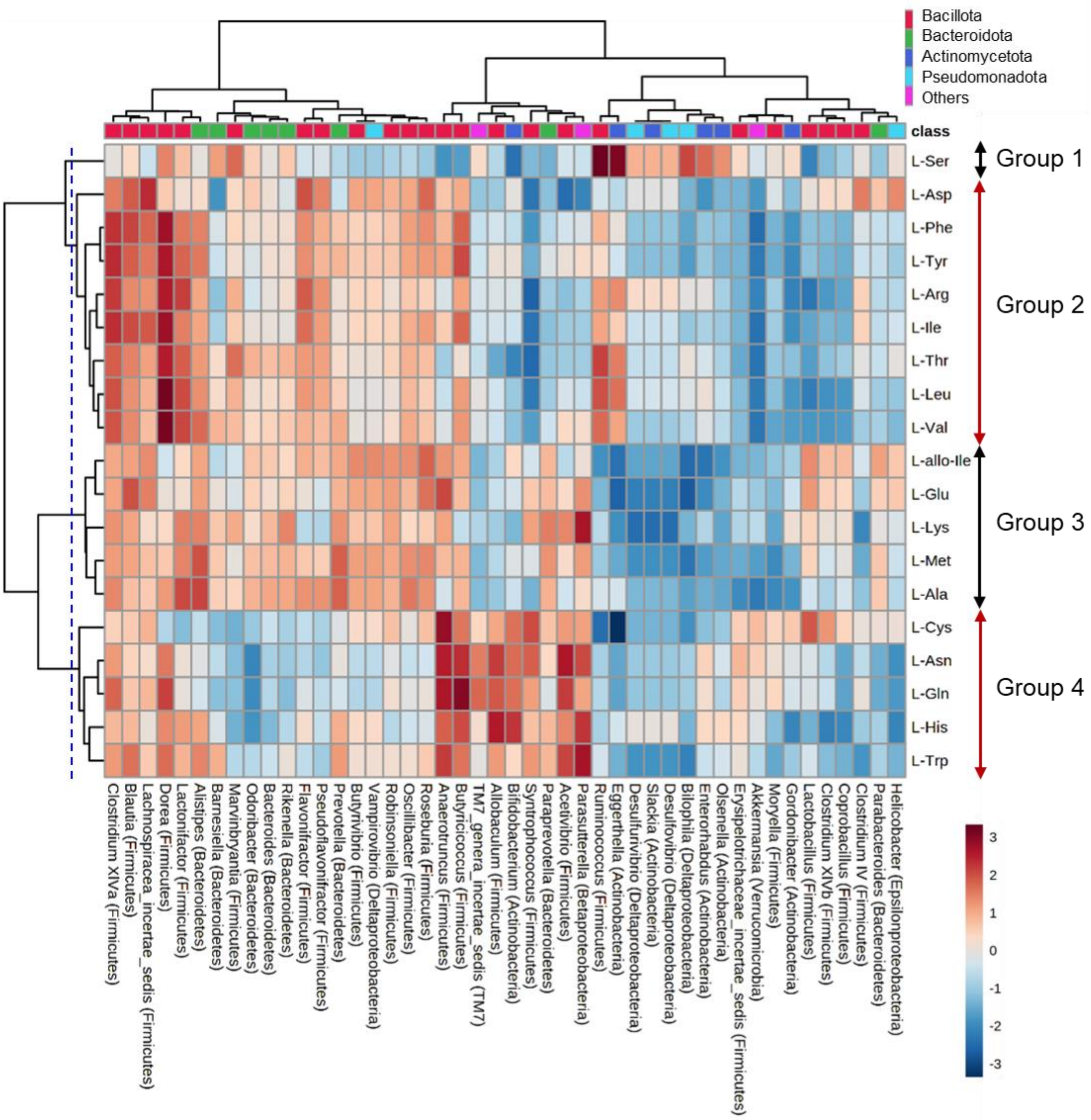

Supplementary Figure 7. Correlation analysis of bacterial composition and metabolome focusing L-amino acids. Chemicals were classified into 4 groups based on threshold shown in blue dot line.

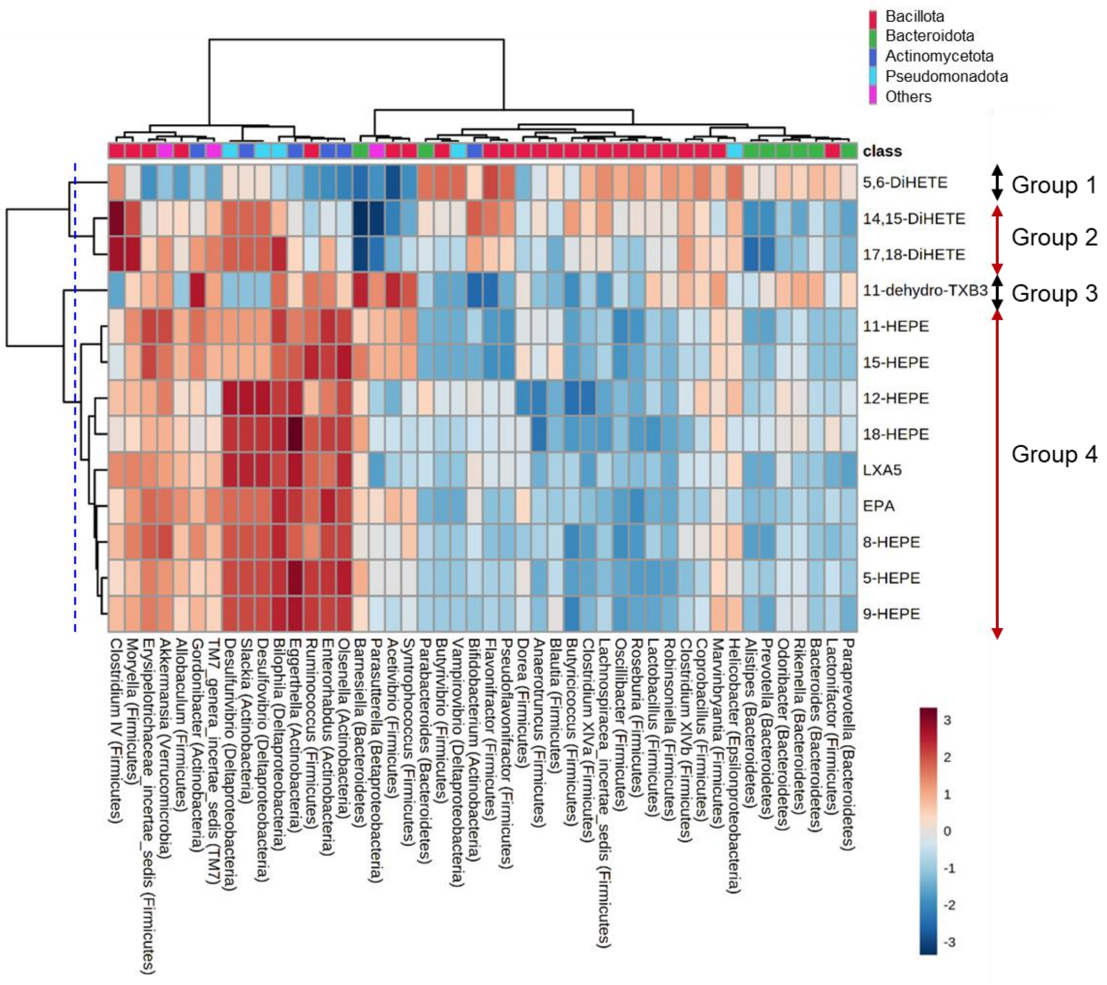

Supplementary Figure 8. Correlation analysis of bacterial composition and metabolome focusing eicosapentaenoic acid (EPA). Chemicals were classified into 4 groups based on threshold shown in blue dot line.

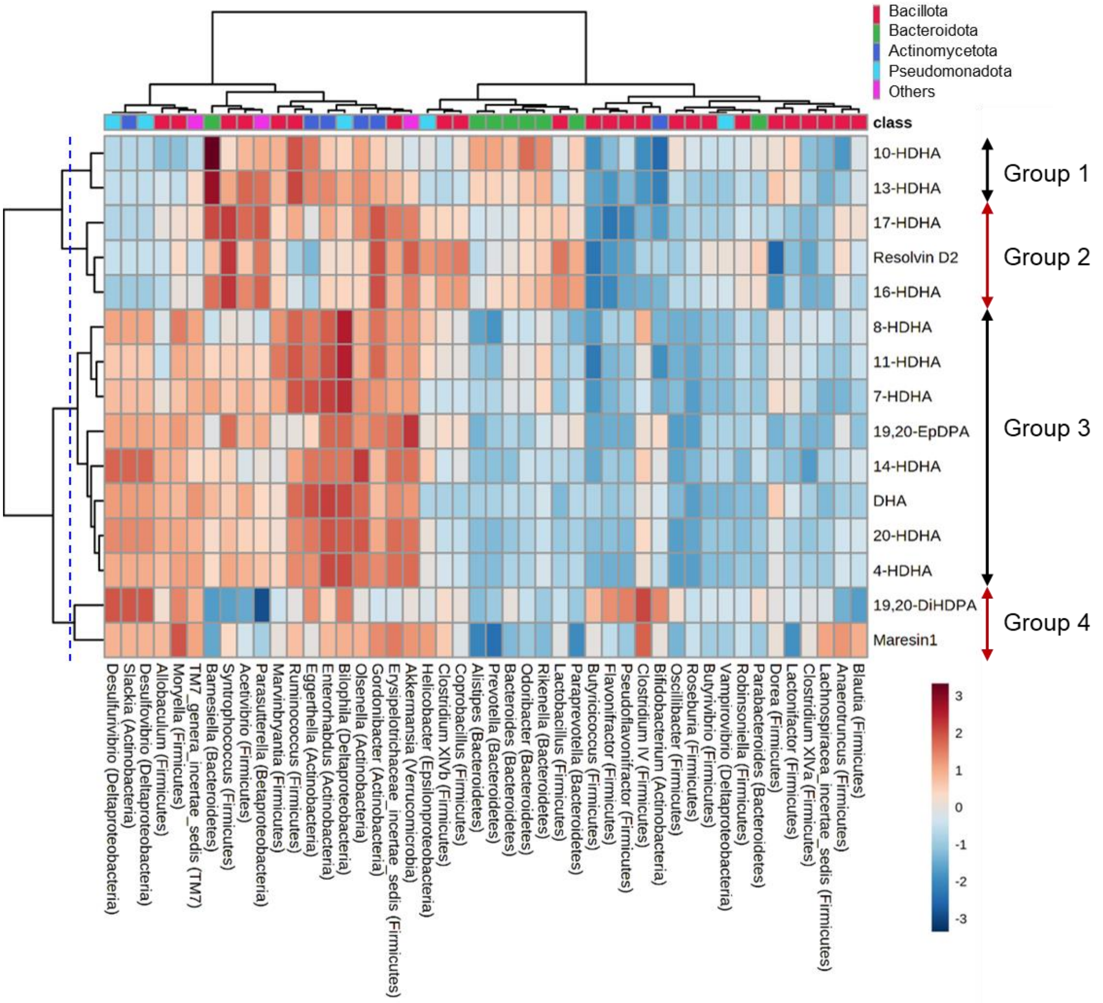

Supplementary Figure 9. Correlation analysis of bacterial composition and metabolome focusing docosahexaenoic acid (DHA). Chemicals were classified into 4 groups based on threshold shown in blue dot line.

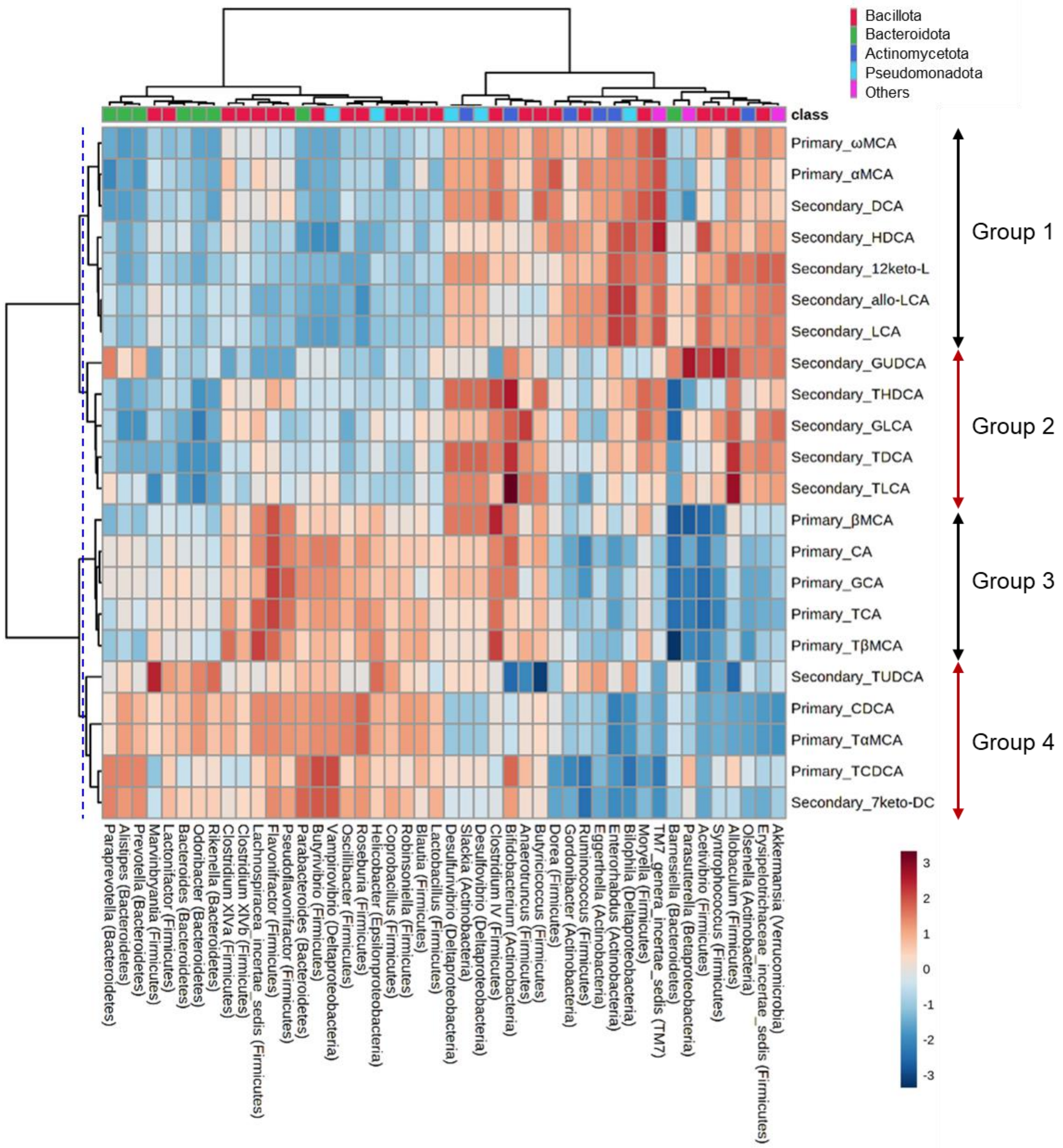

Supplementary Figure 10. Correlation analysis of bacterial composition and metabolome focusing bile acids. Chemicals were classified into 4 groups based on threshold shown in blue dot line.

In this study, TUDCA is classified into primary bile acid as well as Sayin's report in rodents (Cell Metabolism 17, 225-235, 2013)

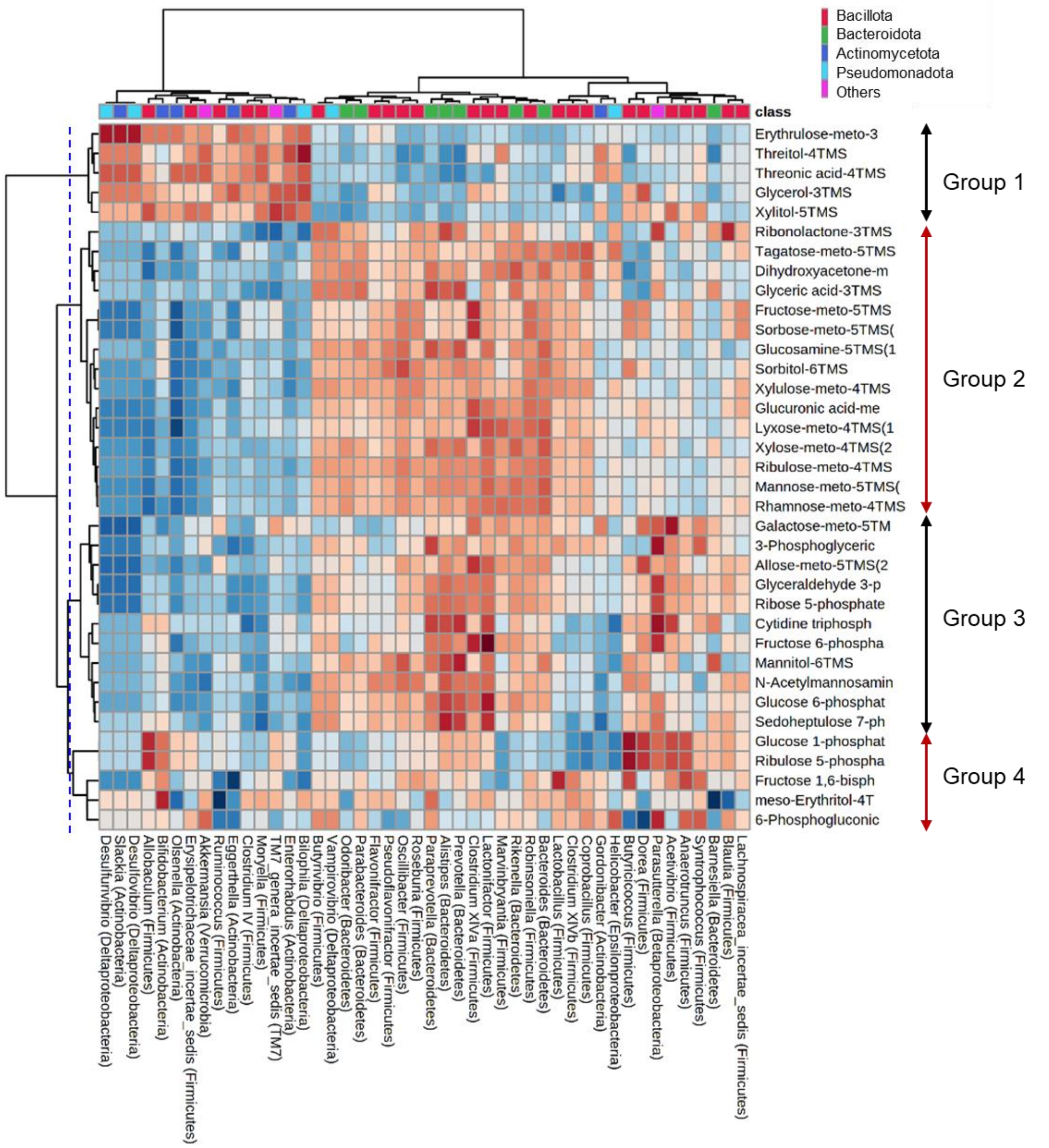

Supplementary Figure 11. Correlation analysis of bacterial composition and metabolome focusing carbohydrate and its conjugates. Chemicals were classified into 4 groups based on threshold shown in blue dot line.

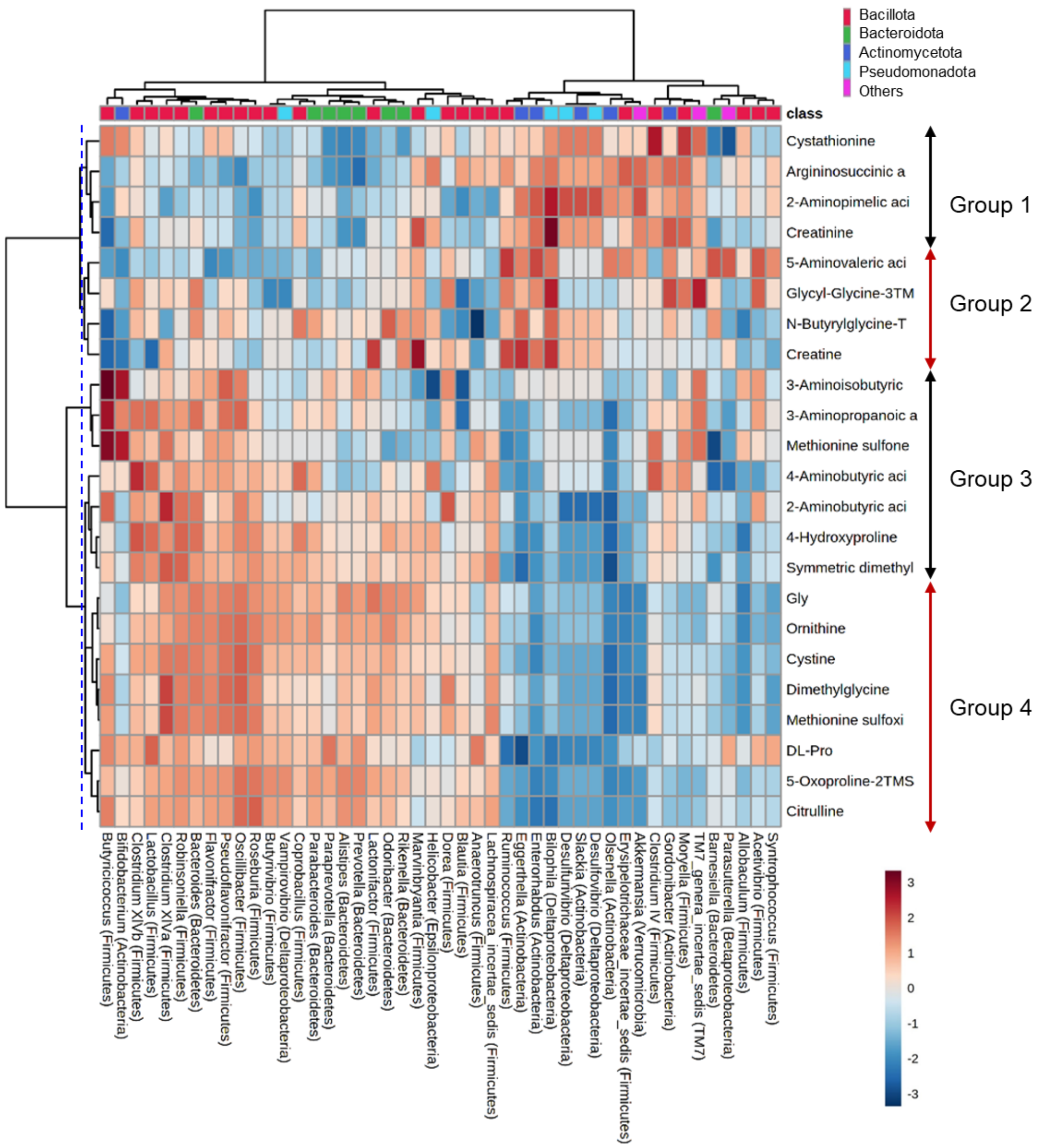

Supplementary Figure 12. Correlation analysis of bacterial composition and metabolome focusing amino acids (excluding basic L- and D-amino acids), peptides and its analogues. Chemicals were classified into 4 groups based on threshold shown in blue dot line.

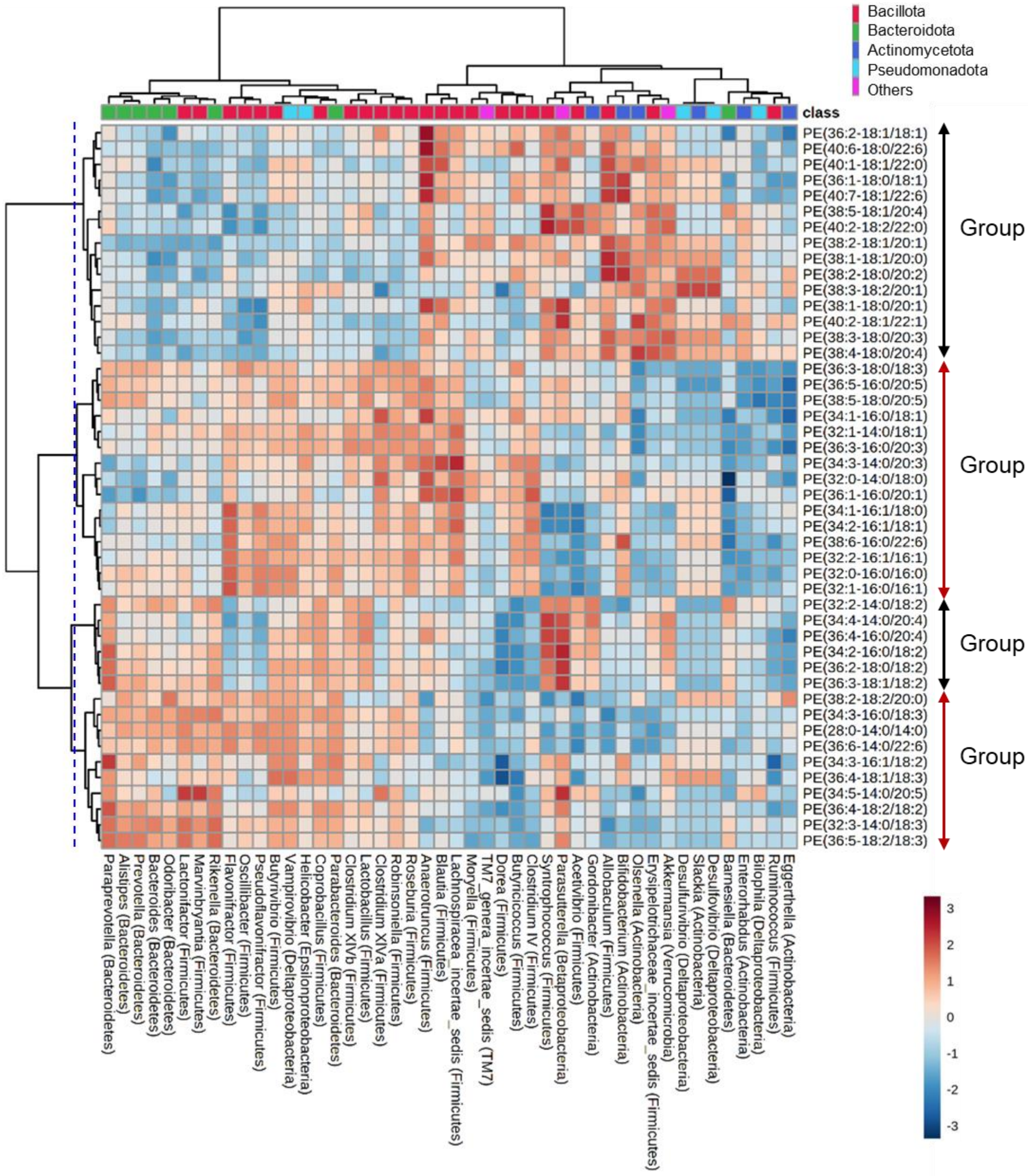

Supplementary Figure 13. Correlation analysis of bacterial composition and metabolome focusing phosphatidylethanolamine (PE). Chemicals were classified into 4 groups based on threshold shown in blue dot line.

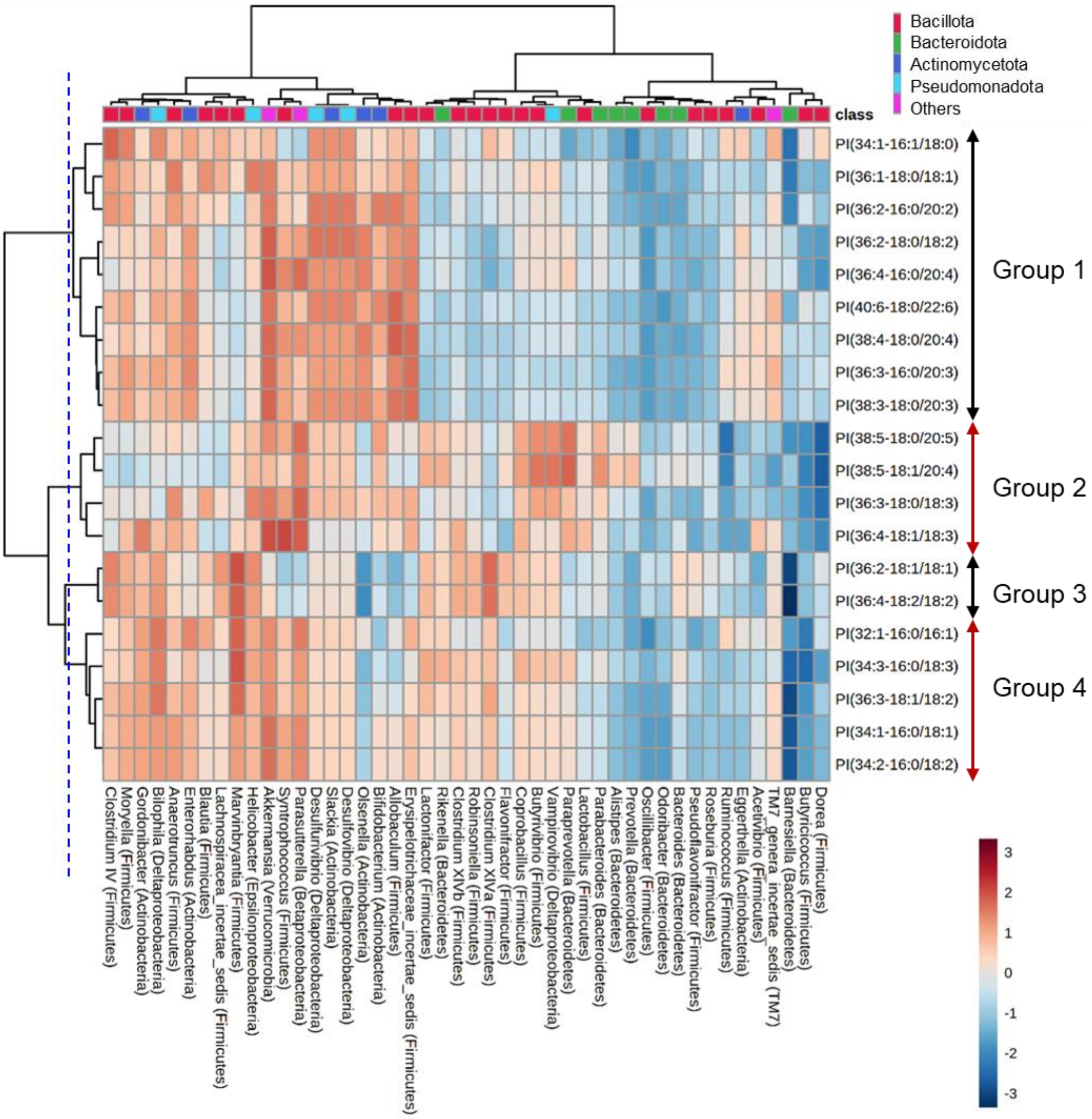

Supplementary Figure 14. Correlation analysis of bacterial composition and metabolome focusing phosphatidylinositol (PI). Chemicals were classified into 4 groups based on threshold shown in blue dot line.
